# Supplementary material for: Generation of functional ciliated cholangiocytes from human pluripotent stem cells
Source: Nat Commun. 2021 Nov 11;12:6504. doi: 10.1038/s41467-021-26764-0 (PMC8586142; doi:10.1038/s41467-021-26764-0)

Supplementary Information

Generation of functional ciliated cholangiocytes from human pluripotent stem cells

Ogawa et al.

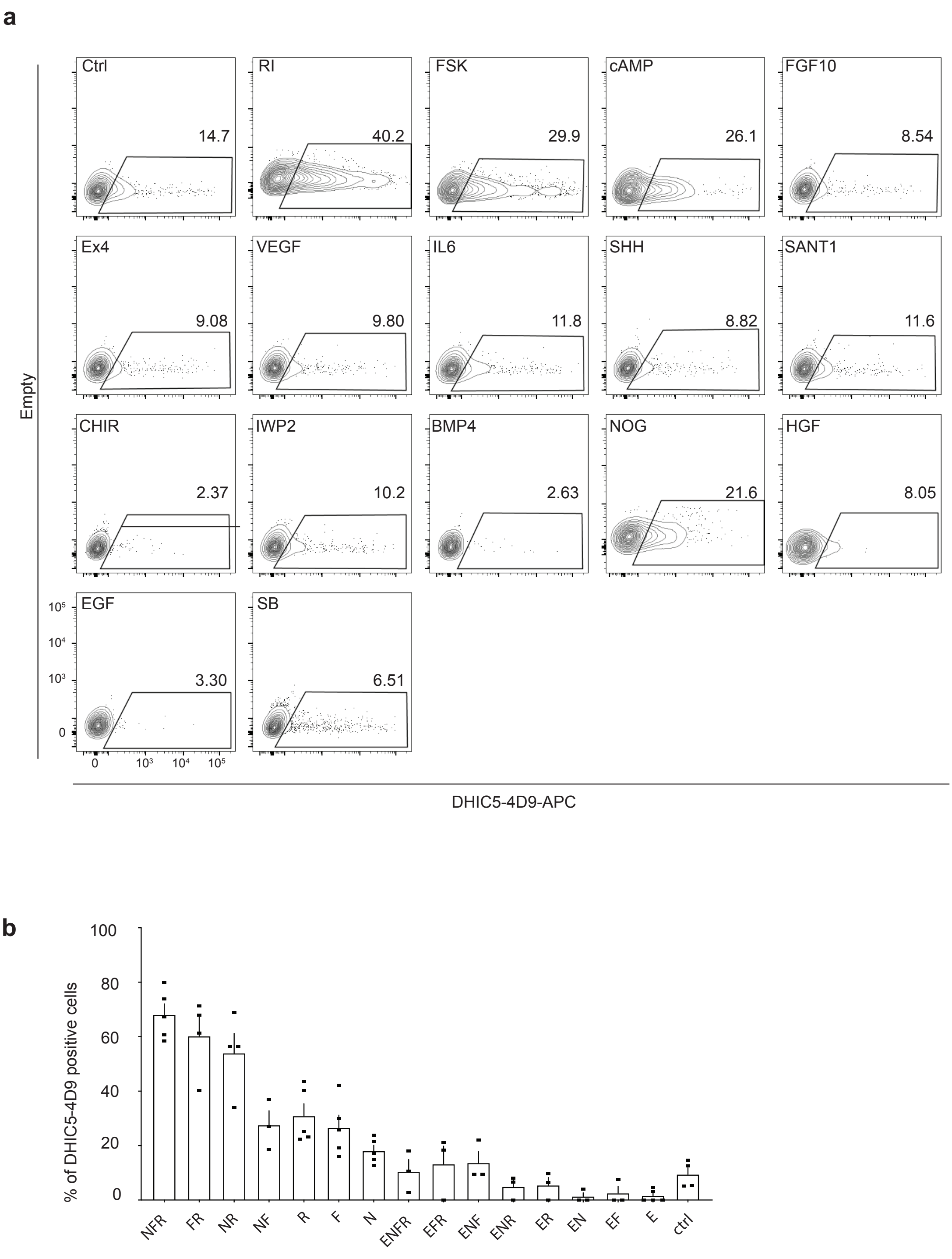

Supplementary Figure 1

Identification of factors which upregulate DHIC5-4D9 expression by Flow cytometry analysis of hPSC-derived cholangiocytes.

(a) Representative flow cytometry analysis screening of the upregulation of DHIC5-4D9 expression in day 43 hPSC derived cholangiocytes after treatment with different indicated cytokines. (b) Quantification of DHIC5-4D9 positive cells in day 43 hPSC-derived cholangiocytes after treatment with different indicated cytokine combinations (n=3-5). Data are represented as mean ± SEM.

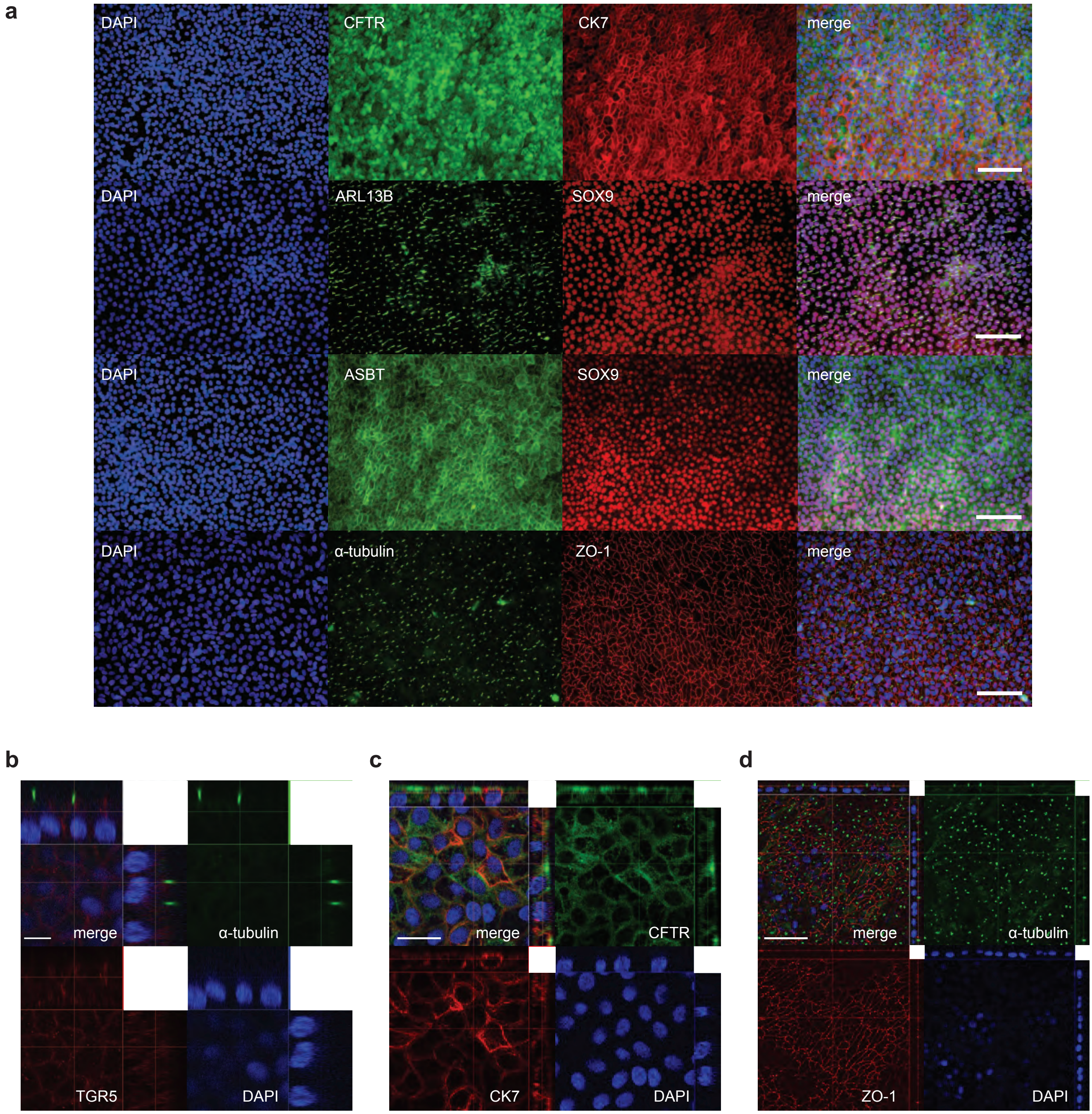

Supplementary Figure 2

Characterization of hPSC-derived cholangiocytes.

(a) Immunostaining images of day49 cholangiocytes demonstrating CFTR (green) and CK7 (red) in the top panel. ARL13B (green) and SOX9 (red) in the second from the top, ASBT (green) and SOX9 (red) in the third panel, acetylated α-tubulin (green) and ZO-1 (red) in the bottom. Scale bar represents 200μm. (b) Side view of immunostaining images of day49 cholangiocytes demonstrating α-tubulin and TGR5. Scale bar represents 5μm. (c) Side view of immunostaining images of day49 cholangiocytes expressing CFTR and CK7. Scale bar represents 25μm. (d) Side view of immunostaining images of day49 cholangiocytes expressing α-tubulin and ZO-1. Scale bar represents 50μm.

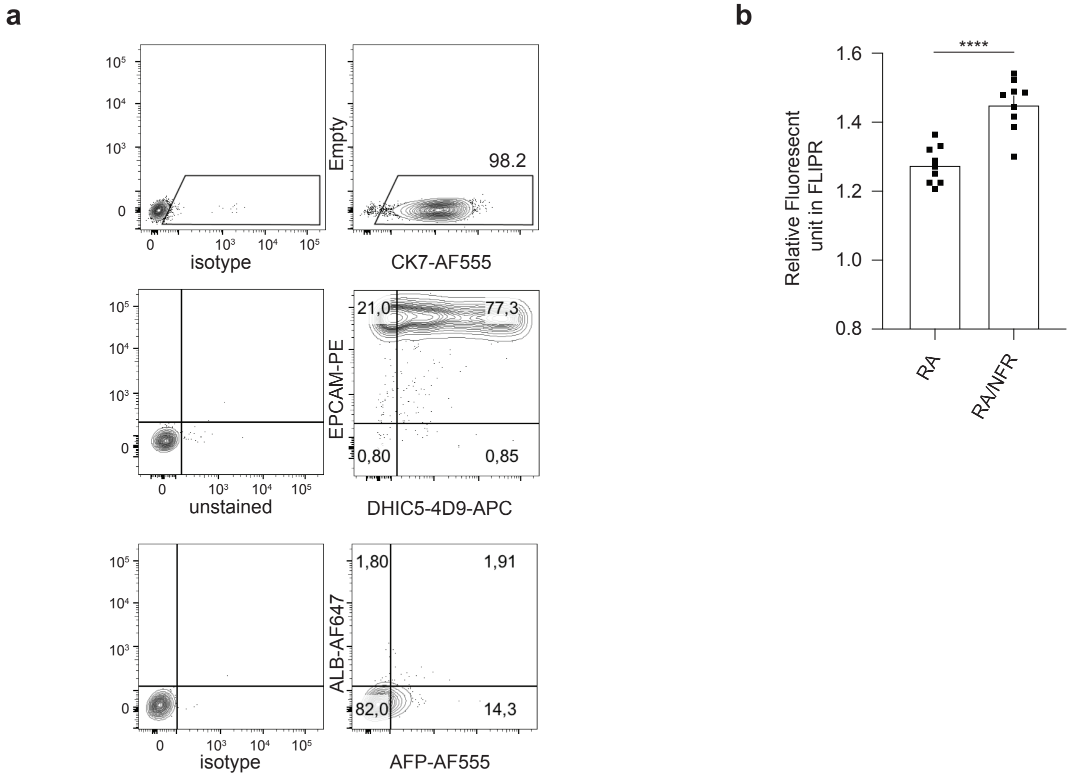

Supplementary Figure 3

Characterization of hPSC-derived cholangiocytes.

(a) Representative flow cytometry analysis showing CK7 (top), EPCAM and DHIC5-4D9 (middle), and ALB and AFP (bottom) positivity in day49 hPSC-derived cholangiocytes. (b) Quantification of the maximum intensity of Apical Chloride Conductance (ACC) in day 37 and day49 cholangiocytes (n=4). Data are represented as mean ± SEM. $^{****}p \leq 0.0001$ two-tailed Student's t-test.

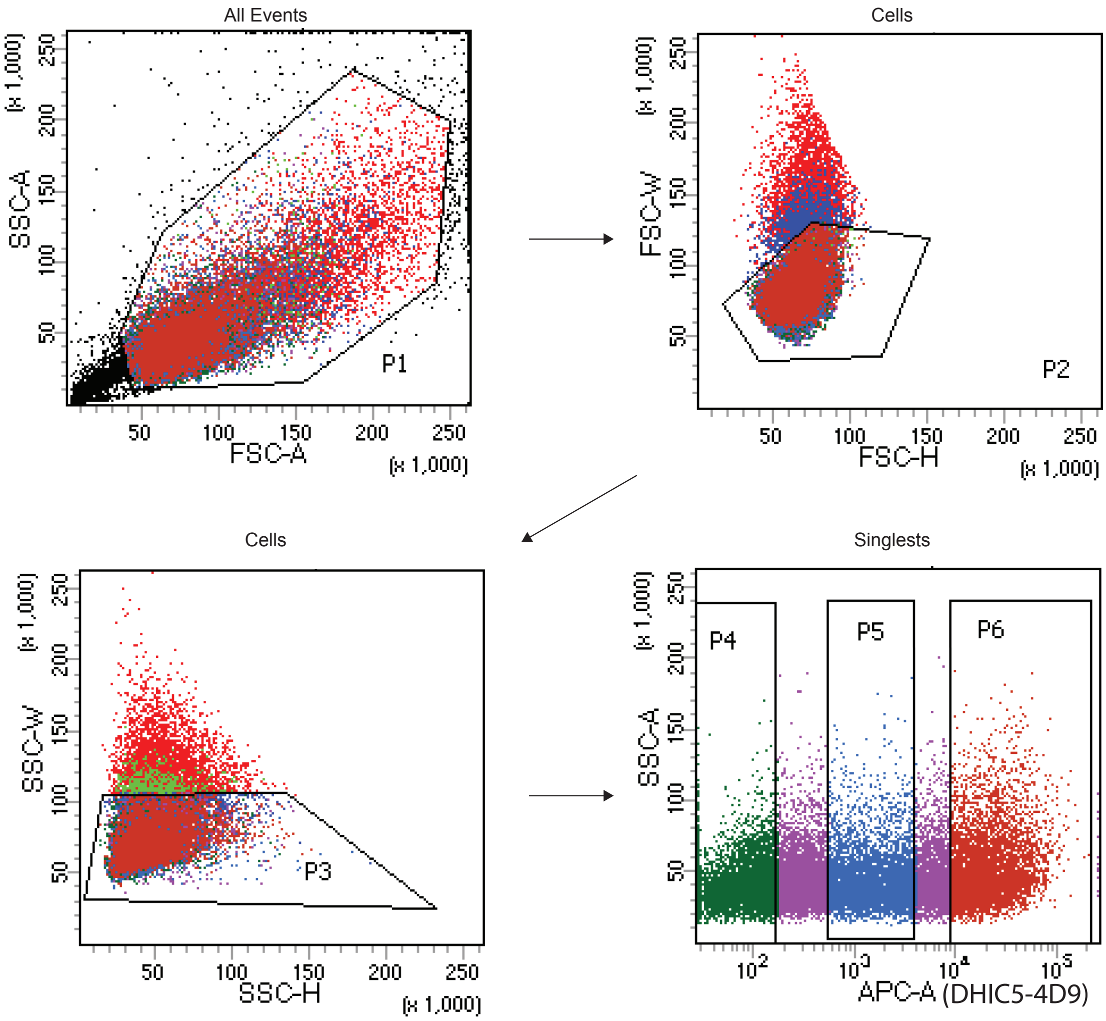

Population	#Events	%Parent	%Total
All Events	40,555	####	100.0
P1	30,000	74.0	74.0
P2	24,812	82.7	61.2
P3	25,436	84.8	62.7
P2 AND P3	24,327	81.1	60.0
P4	6,733	27.7	16.6
P5	4,793	19.7	11.8
P6	6,122	25.2	15.1

Supplementary Figure 4
Cell gating strategy for flow cytometry analysis and cell sorting experiments. APC-A was for DHIC5-4D9. Day49 H9 derived cholangiocytes were sorted for negative (P4) and positive (P6) populations (n=3).

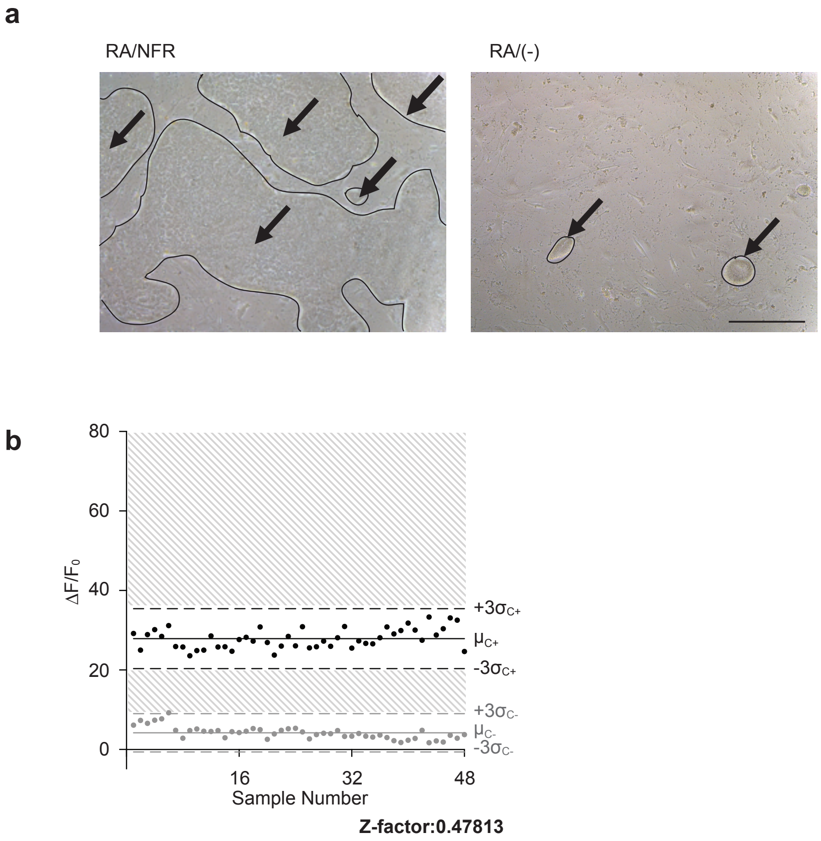

Supplementary Figure 5

Characterization of hPSC-derived cholangiocytes.

(a) Microphotographic images show the bright field images of day 49 cholangiocytes in the presence of indicated treatments. Line traces and arrows indicate the cholangiocyte colonies. Scale bar represents 500µm. (b) The Z factors show the quality of the FLIPR assay measuring Apical Chloride Conductance (ACC) in 96 well plate with hPSC-derived cholangiocytes at day37.

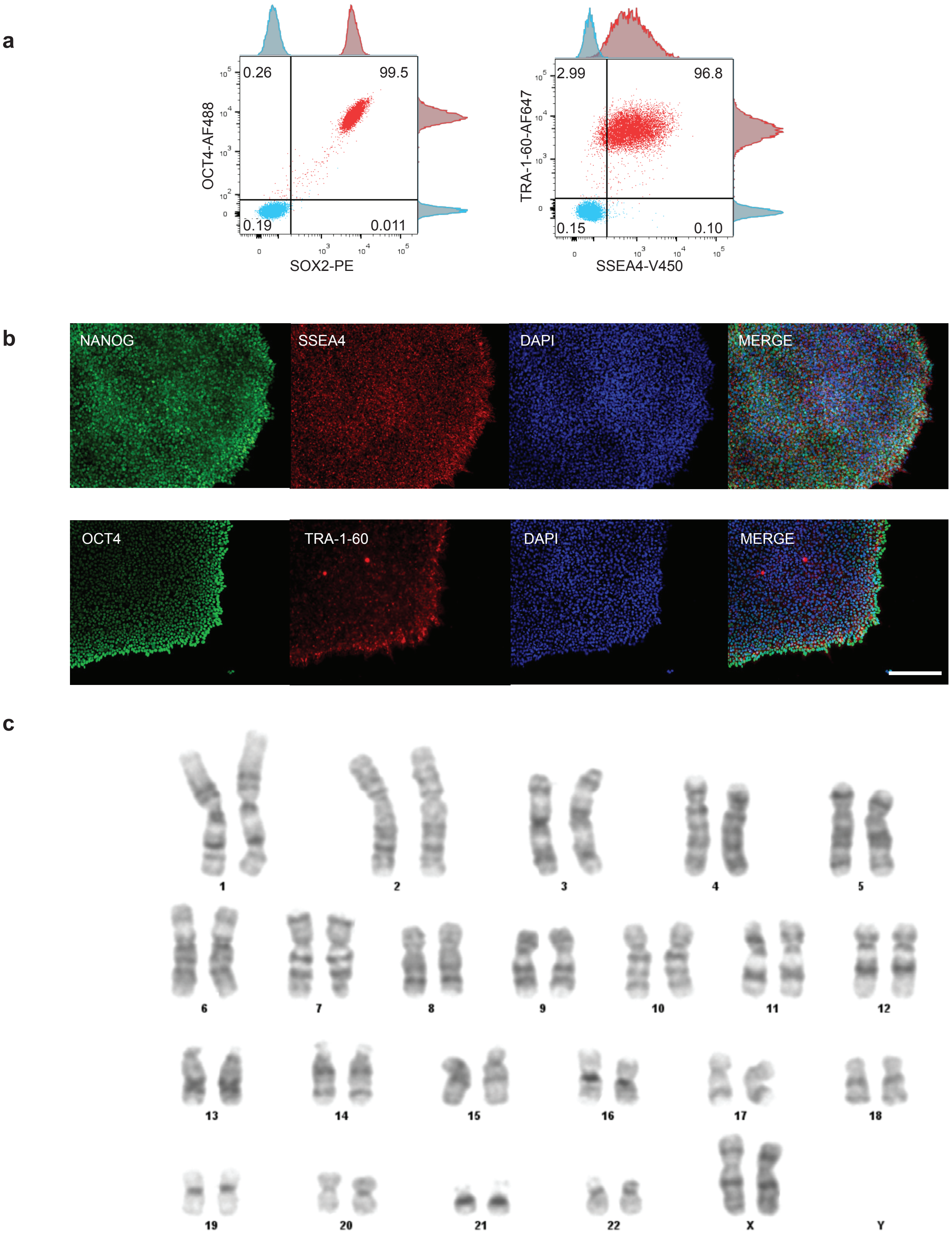

Supplementary Figure 6
Characterization of WT01 (wild type) iPSCs.
(a) Representative flow cytometric analysis showing OCT4, SOX2, TRA-1-60, and SSEA4 positivity. (b) Immunostaining images of iPS colony demonstrating pluripotent markers NANOG, and SSEA4 in upper panel, OCT4 and TRA-1-60 in the lower panel. Scale bar represents 400µm. (c) G-banding analysis shows normal karyotype.

a

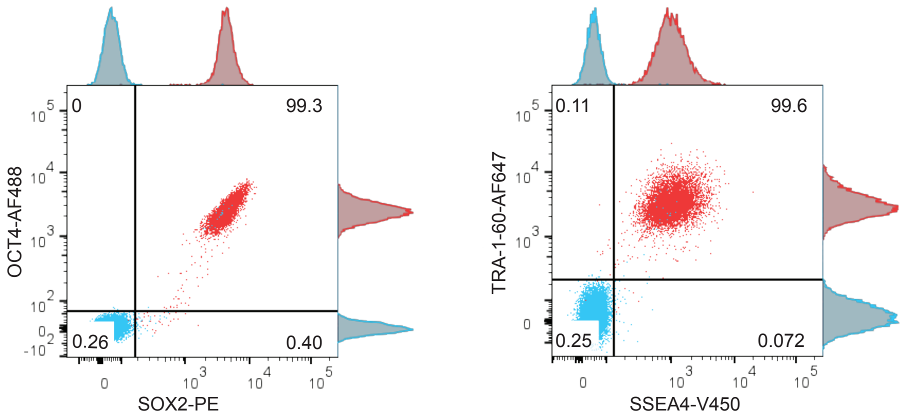

b

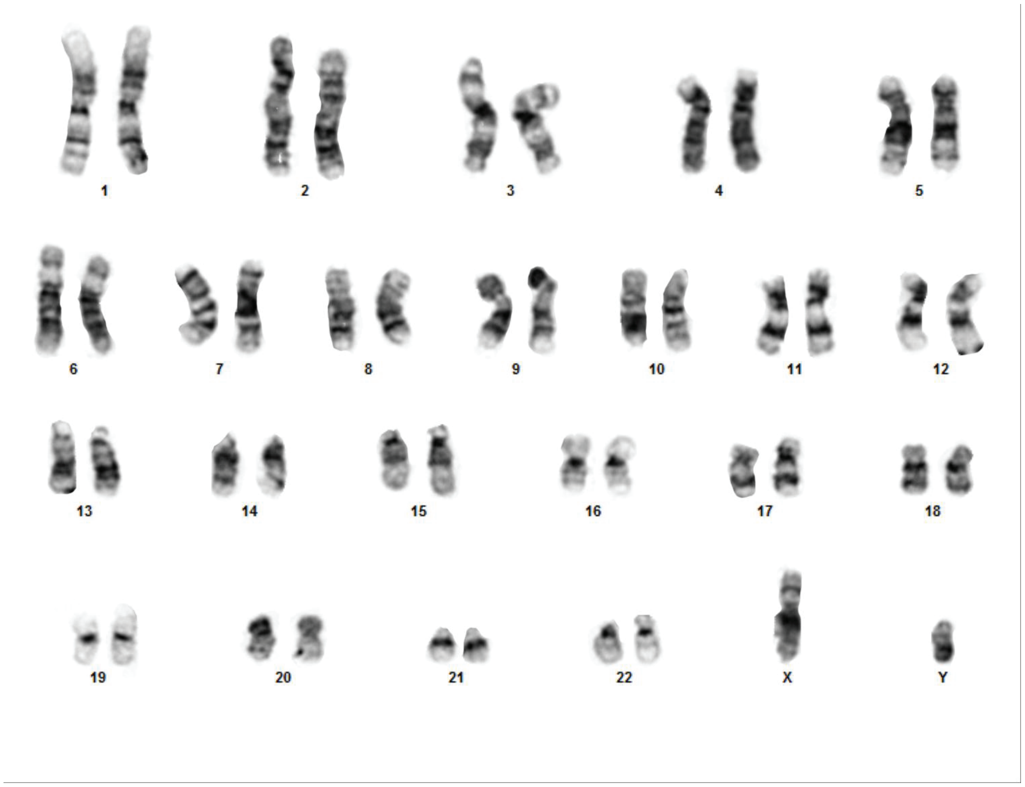

Supplementary Figure 7
Characterization of CF01 (F508del) iPSCs.
(a) Representative flow cytometric analysis showing OCT4, SOX2, TRA-1-60, and SSEA4 positivity. (b) G-banding analysis shows normal karyotype.

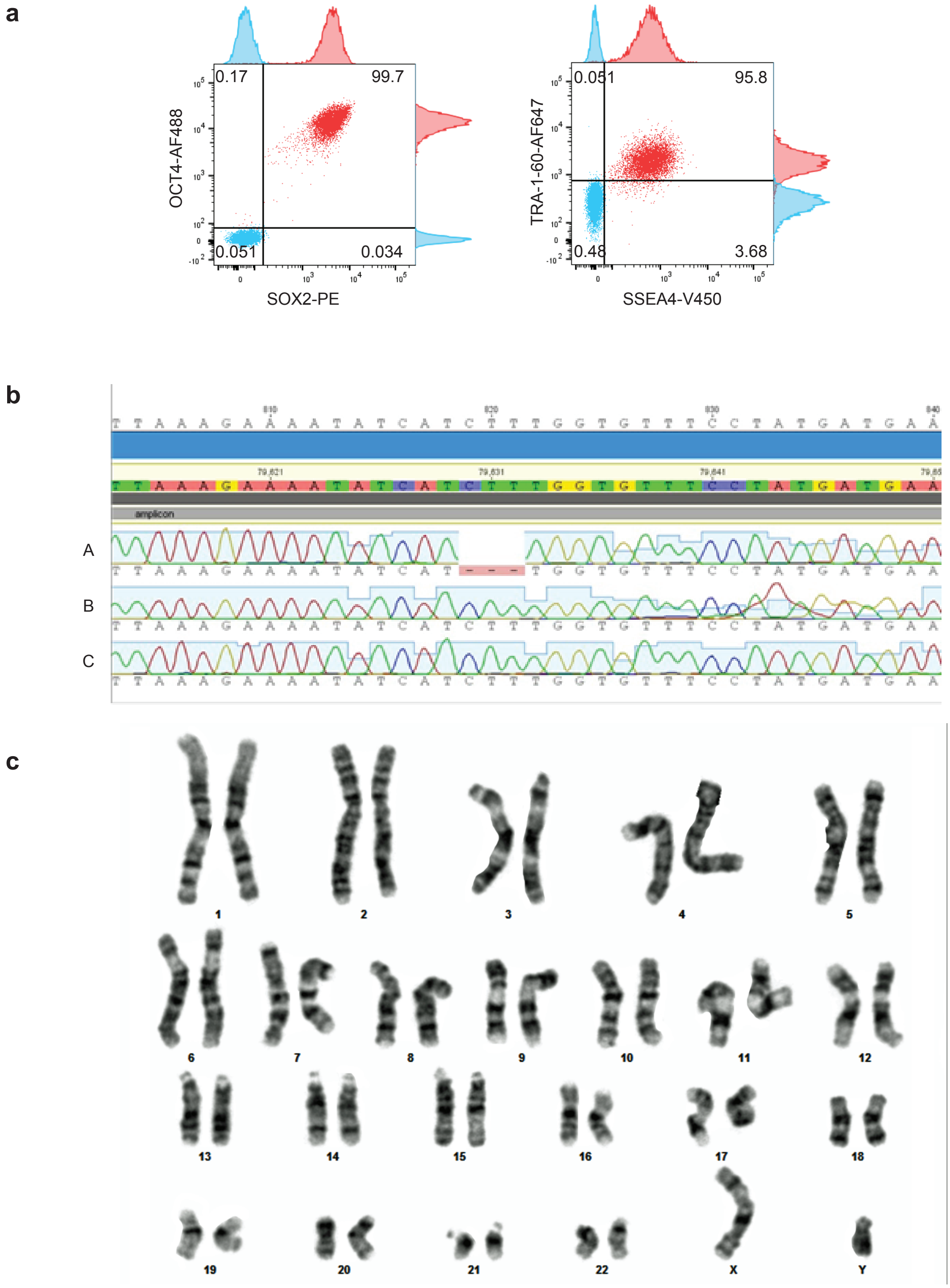

Supplementary Figure 8

Characterization of CF01MC (F508del mutation corrected) iPSCs.

(a) Representative flow cytometric analysis showing OCT4, SOX2, TRA-1-60, and SSEA4 positivity. (b) Sequencing of CFTR region of interest from (A) Genomic DNA extracted from CF01 showing cells homozygous for F508del. (B) Genomic DNA extracted from CF01MC corrected line showing cells homozygous for correction to wildtype. (C) Sequencing of (B) after TOPO cloning the CFTR PCR product showing the complete corrected sequence. (c) G-banding analysis shows normal karyotype.

a

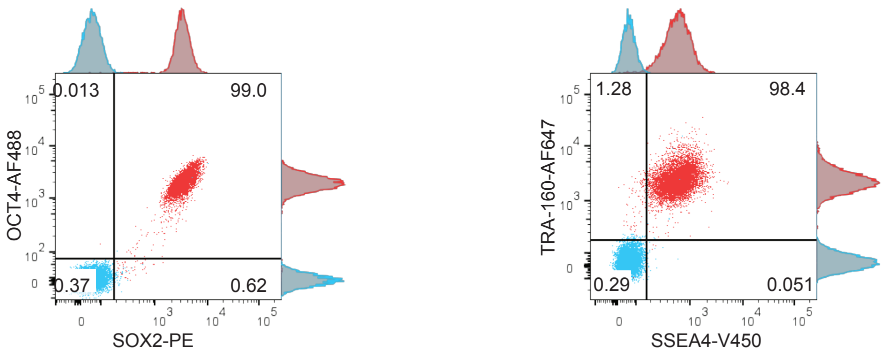

b

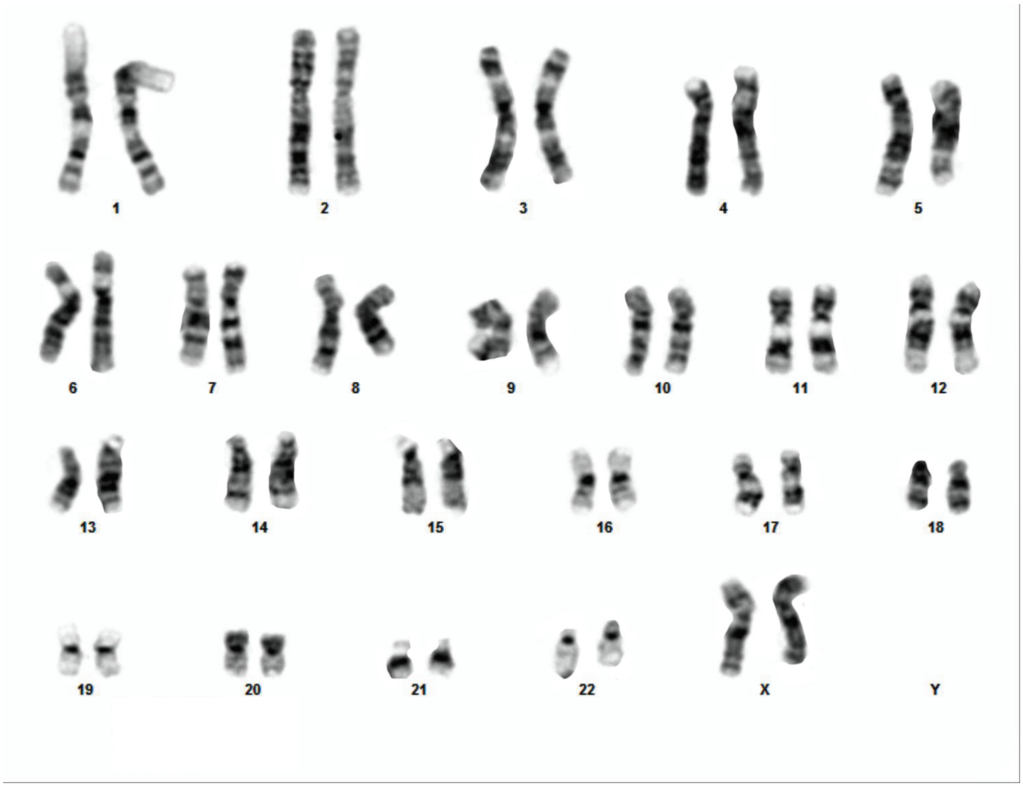

Supplementary Figure 9
Characterization of CF02 (F508del) iPSCs.
(a) Representative flow cytometric analysis showing OCT4, SOX2, TRA-1-60, and SSEA4 positivity. (b) G-banding analysis shows normal karyotype.

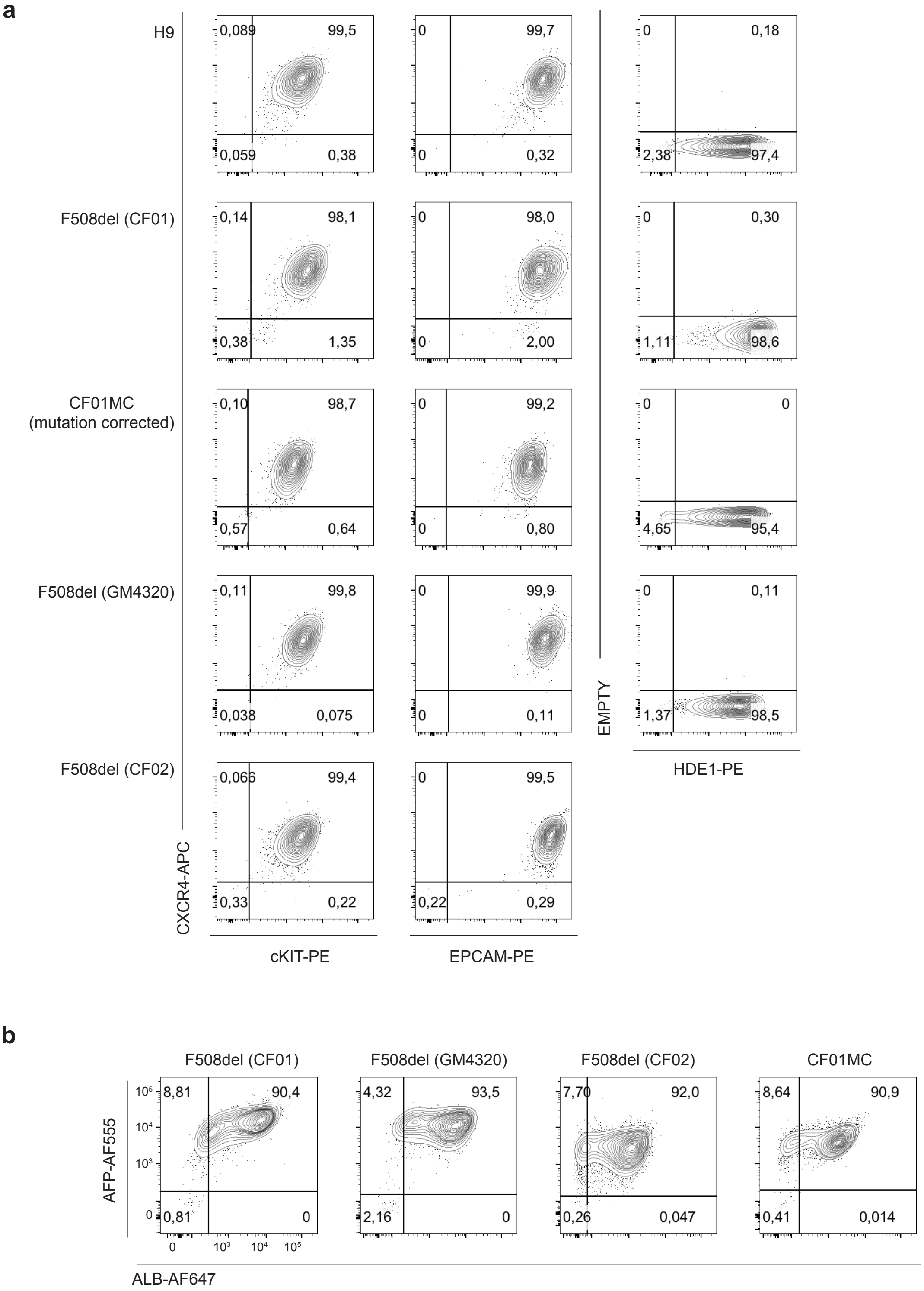

Supplementary Figure 10
Characterization of hPSC-derived endoderm and hepatoblasts.
(a) Representative flow cytometry analysis of day9 hPSC-derived endoderm from different cell lines. (b) Representative flow cytometry analysis showing ALB and AFP positivity in day27-33 CF patients-derived hepatoblasts.

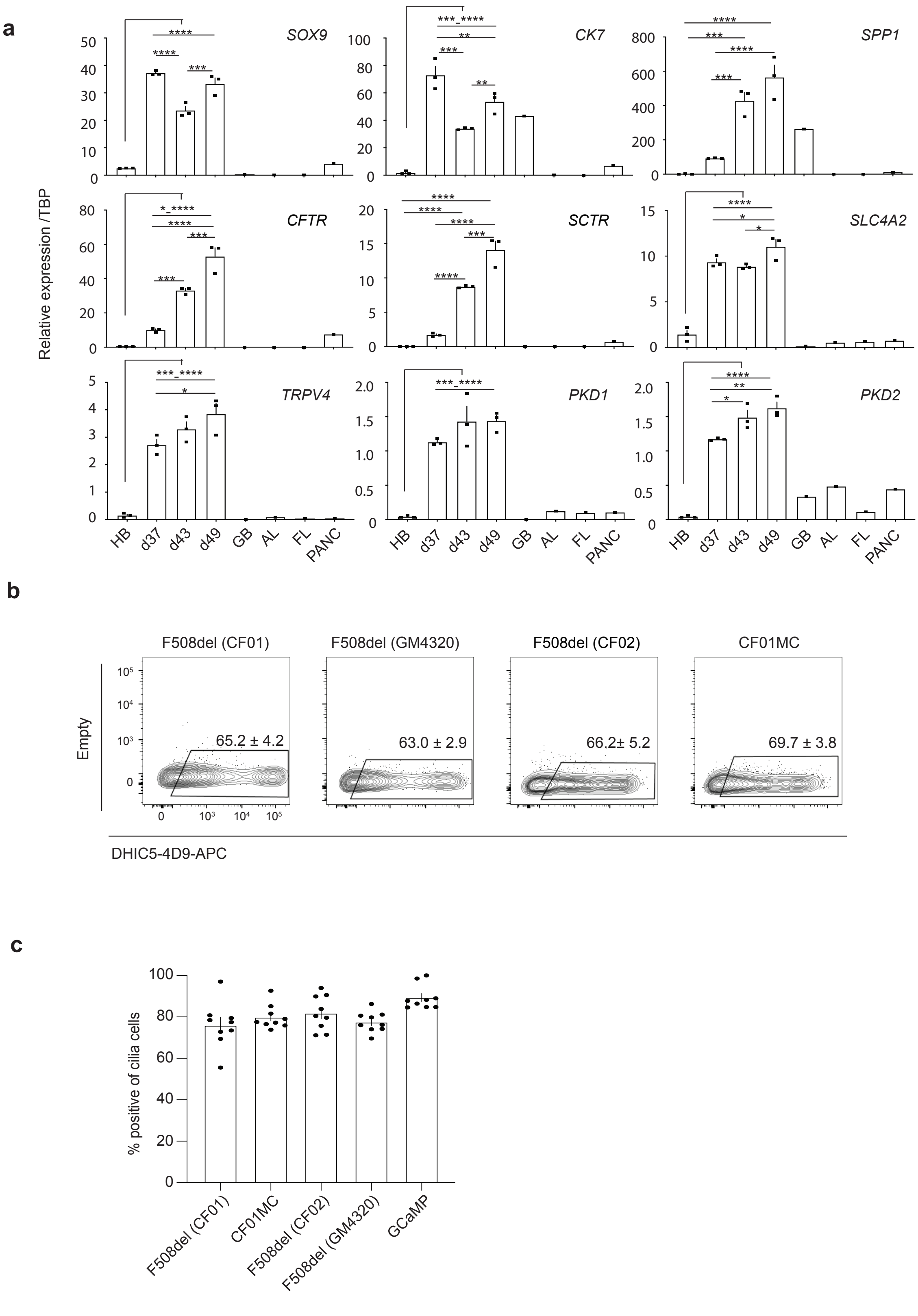

Supplementary Figure 11

Supplementary Figure 11
Characterization of hPSC-derived cholangiocytes.
(a) qPCR analysis shows the expression of indicated genes in different stages of the CF01a (F508del) cholangiocyte differentiation. HB: hepatoblasts, GB: gall bladder, AL: adult liver, FL: fetal liver, PANC: pancreas (n=3). Data are represented as mean ± SEM. $^{*}p \leq 0.05$, $^{**}p \leq 0.01$, $^{***}p \leq 0.001$, $^{****}p \leq 0.001$ one-way ANOVA. (b) Representative flow cytometry analysis showing DHIC5-4D9 positivity in day49 hPSC derived cholangiocytes from day49-55 CF iPSC derived cholangiocytes (n=3-5). Data are represented as mean ± SEM. (c) Quantification of cilia positive cells in day49 from different hPSC derived cholangiocytes (n=3). Data are represented as mean ± SEM.

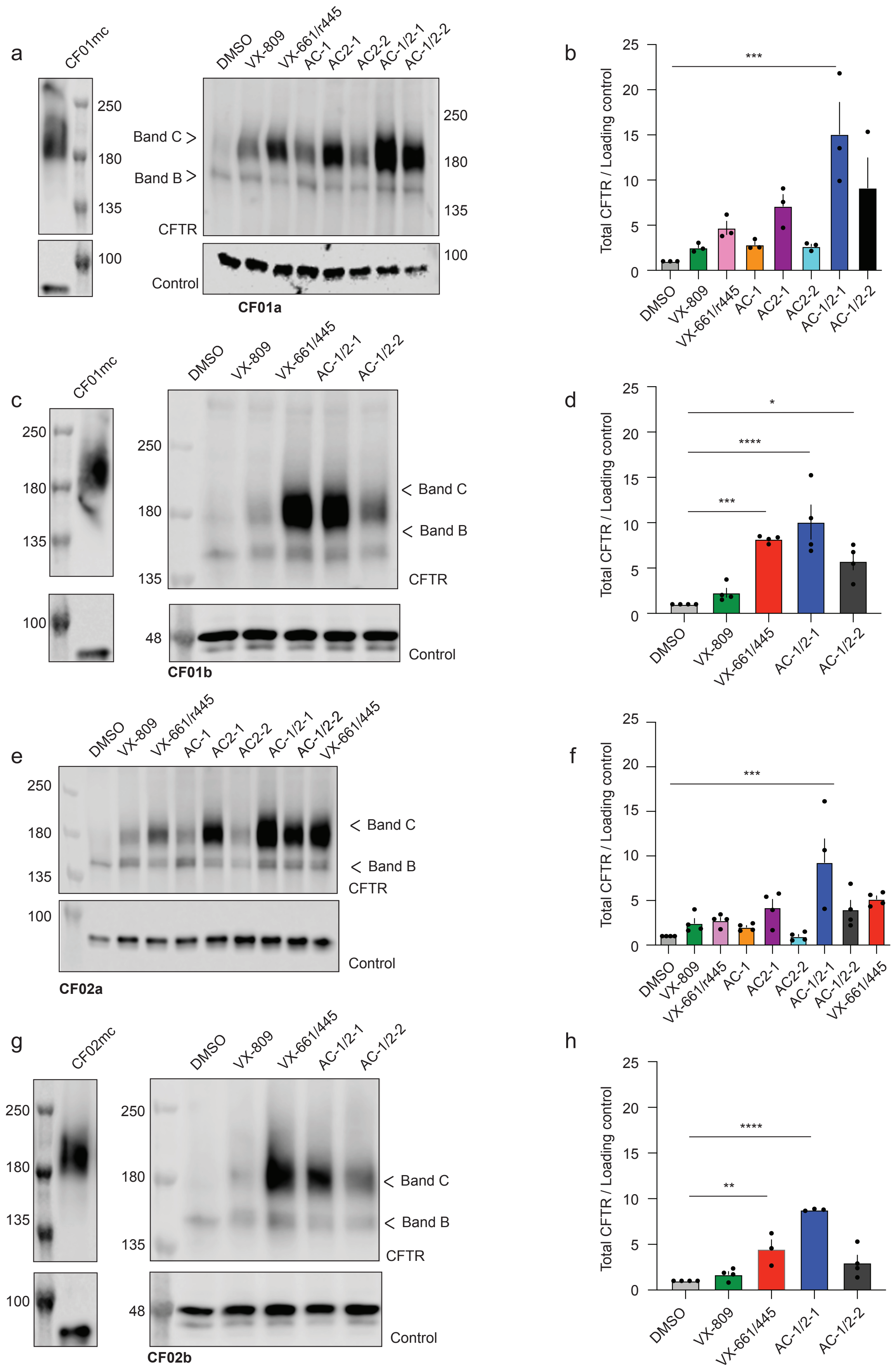

Supplementary Figure 12

Supplementary Figure 12
Drug response in CF iPSC-derived cholangiocyte.

(a) Western blotting shows the immature (B) and mature (C) glycosylated CFTR bands after treatment with CFTR modulators in day49 F508del (CF01a) cholangiocyte (right) and CF01MC cholangiocyte (left). (b) Quantification of western blotting of the ratio of mature glycosylated CFTR protein with different CFTR modulators in day49 CF01a patient's iPSC-derived cholangiocytes (n=3). (c) Western blotting shows the immature (B) and mature (C) glycosylated CFTR bands after treatment with CFTR modulators in day49 F508del (CF01b) cholangiocyte (right) and CF01MC cholangiocyte (left). (d) Quantification of western blotting of the ratio of mature glycosylated CFTR protein with different CFTR modulators in day49 CF01b patient's iPSC-derived cholangiocytes (n=4). (e) Western blotting shows the immature and mature glycosylated CFTR bands after treatment with CFTR modulators in day49 F508del (CF02a) cholangiocyte. (f) Quantification of western blotting of the ratio of mature glycosylated CFTR protein with different CFTR modulators in day49 CF02a patient's iPSC-derived cholangiocytes (n=4). (g)Western blotting shows the immature (B) and mature (C) glycosylated CFTR bands after treatment with CFTR modulators in day49 F508del (CF02b) cholangiocyte (right) and CF02MC cholangiocyte (left). (h) Quantification of western blotting of the ratio of mature glycosylated CFTR protein with different CFTR modulators in day49 CF02b patient's iPSC-derived cholangiocytes (n=4). One-way ANOVA. $^{*}p \leq 0.05$, $^{**}p \leq 0.01$, $^{***}p \leq 0.001$, $^{****}p \leq 0.0001$

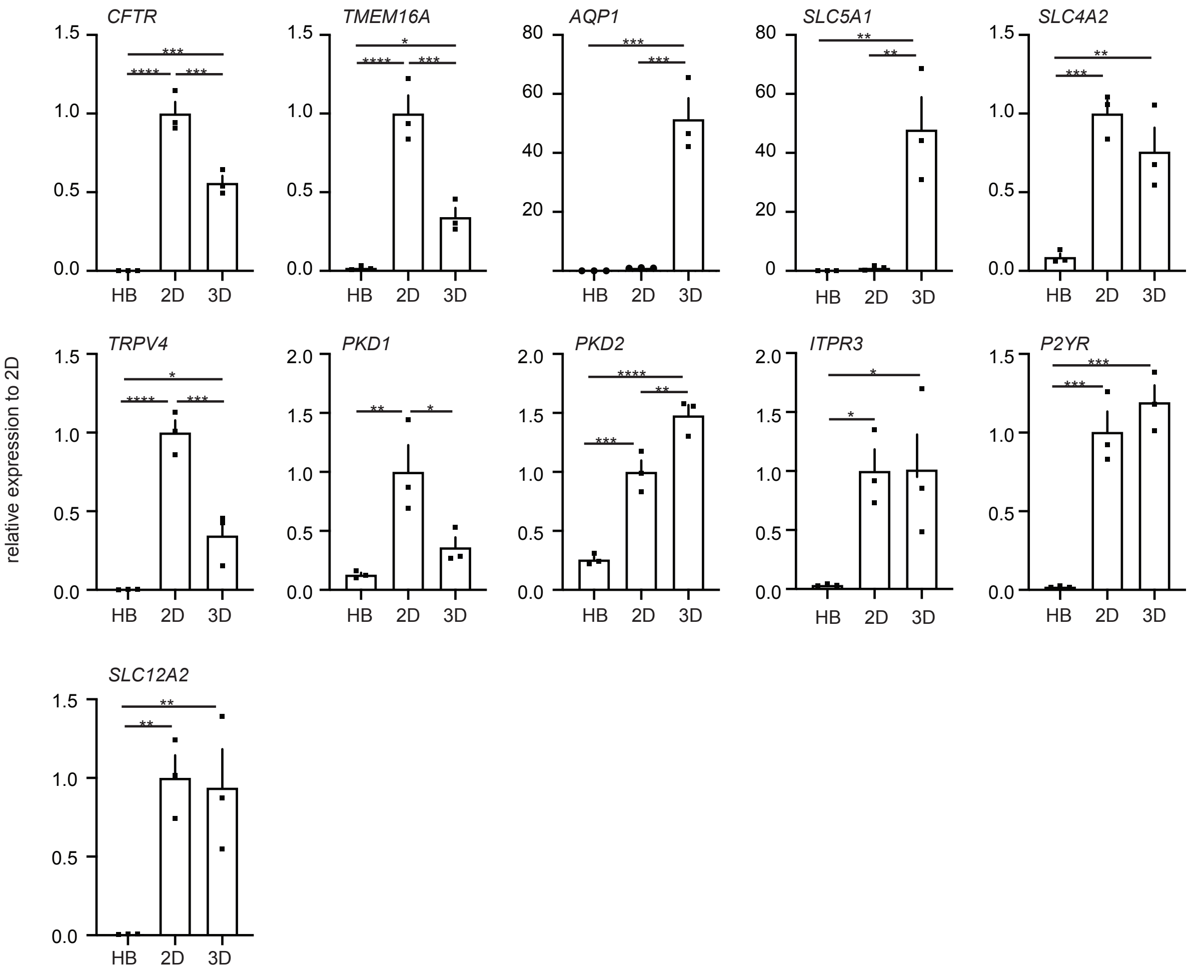

Supplementary Figure 13

RT-qPCR based expression analysis of indicated genes following 6 days culture with indicated conditions from H9-derived cholangiocyte. HB: day 27 H9 derived hepatoblasts, 2D: day55 monolayer condition, 3D cyst: day55 liquid culture condition, $^{*}p \leq 0.05$, $^{**}p \leq 0.01$, $^{***}p \leq 0.001$, $^{****}p \leq 0.0001$ one-way ANOVA. Data are represented as mean ± SEM (n=3).

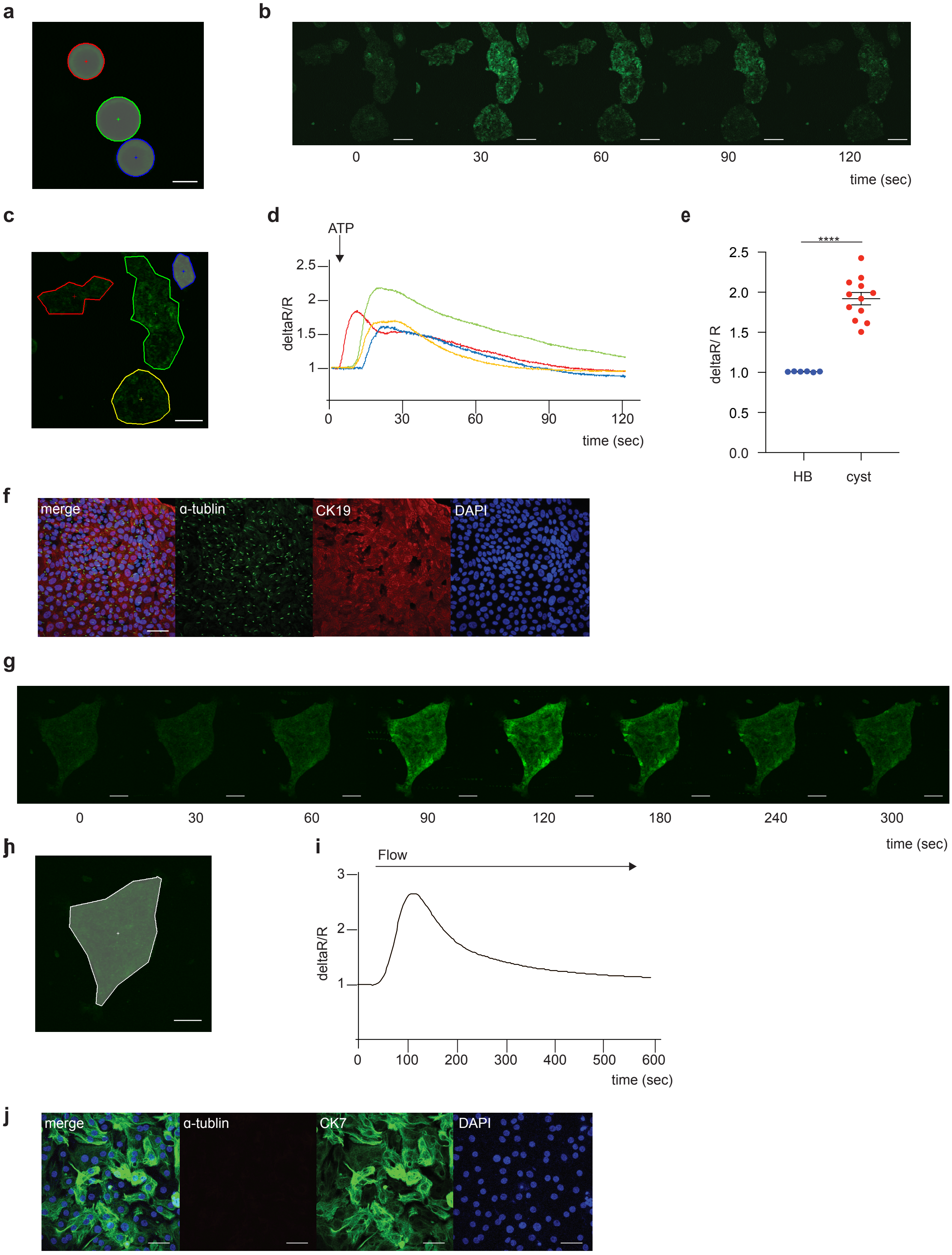

Supplemetary Figure 14

Supplementary Figure 14
Intracellular Calcium release in ciliated hPSCs derived cholangiocyte.
(a) The region of interest (ROI)s correspond to GCaMP derived cholangiocyte cysts (3D) in main Figure 6a. Scale bar represents 100 µm. (b) Representative time lapse images of calcium influx in plated down 3D cholangiocyte cysts from GCaMP hESC in response to ATP. Scale bar represents 100µm. (c) The ROIs mark the GCaMP derived plated down cholangiocyte cysts. Scale bar represents 100 µm. (d) Representative traces showing the intracellular calcium release in plated down cholangiocyte cysts from GCaMP hESC in the response to ATP. The colors of the traces correspond to those for the ROIs in (c). (e) Quantification of maximum fluorescent intensity representing intracellular calcium release in plated down 3D hepatoblast and 3D cholangiocyte cysts from GCaMP hESC in the response to ATP (HB n=4, chol n=3). Data are represented as mean ± SEM. $^{****}p \leq 0.0001$ two-tailed Student's t-test. (f) Representative microscopic images of plate down 3D cholangiocyte cysts (GCaMP hESC) exhibiting primary cilia (green) and CK19 (red). Scale bar represents 50µm. (g) Representative time lapse images of calcium influx in plated down 3D cholangiocyte cysts from GCaMP hESC in the response to flow. Scale bar represents 100µm. (h) The ROIs mark the GCaMP derived plated down cholangiocyte cysts. Scale bar represents 100 µm. (i) Representative trace of the intra cellular calcium release in plated down 3D cholangiocyte cysts from GCaMP hESC in the response to flow correspond to the ROI in (h). (j) Representative microscopic images of non-ciliated CK7 positive cholangiocytes derived from GCaMP hESC. Scale bar represents 50µm.

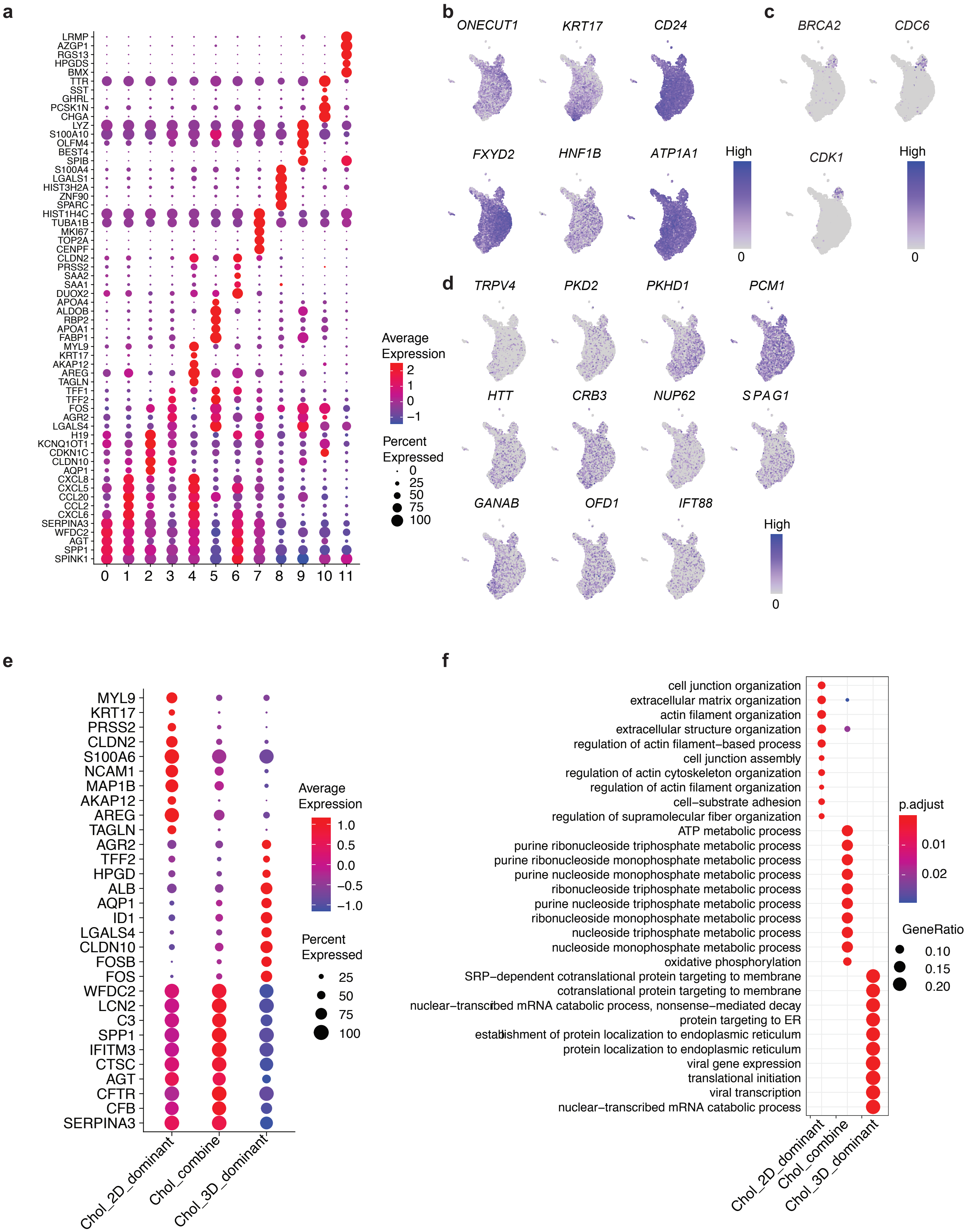

Supplemetary Figure 15

Supplementary Figure 15

Global gene analysis of hPSC-derived cholangiocytes in monolayer (2D) and cyst (3D).

(a) Dot plots displaying the top 5 genes in the 12 clusters of hPSC-derived cholangiocyte. (b) UMAP plots showing the expression of core cholangiocyte genes. (c) UMAP plots showing the expression of cell cycle genes including *BRCA2*, *CDC6*, and *CDK1*. (d) UMAP plots showing the expression of primary cilia target genes. (e) Dot plots displaying the top 10 genes of monolayer (2D) dominant, cyst (3D) dominant, and combine (2D and 3D) populations. Size of the dot represents proportion of the population that express each gene. Color indicates the average expression level. (f) Gene ontology analysis (biological process) of monolayer (2D) dominant, cyst (3D) dominant, and combine (2D and 3D) populations.

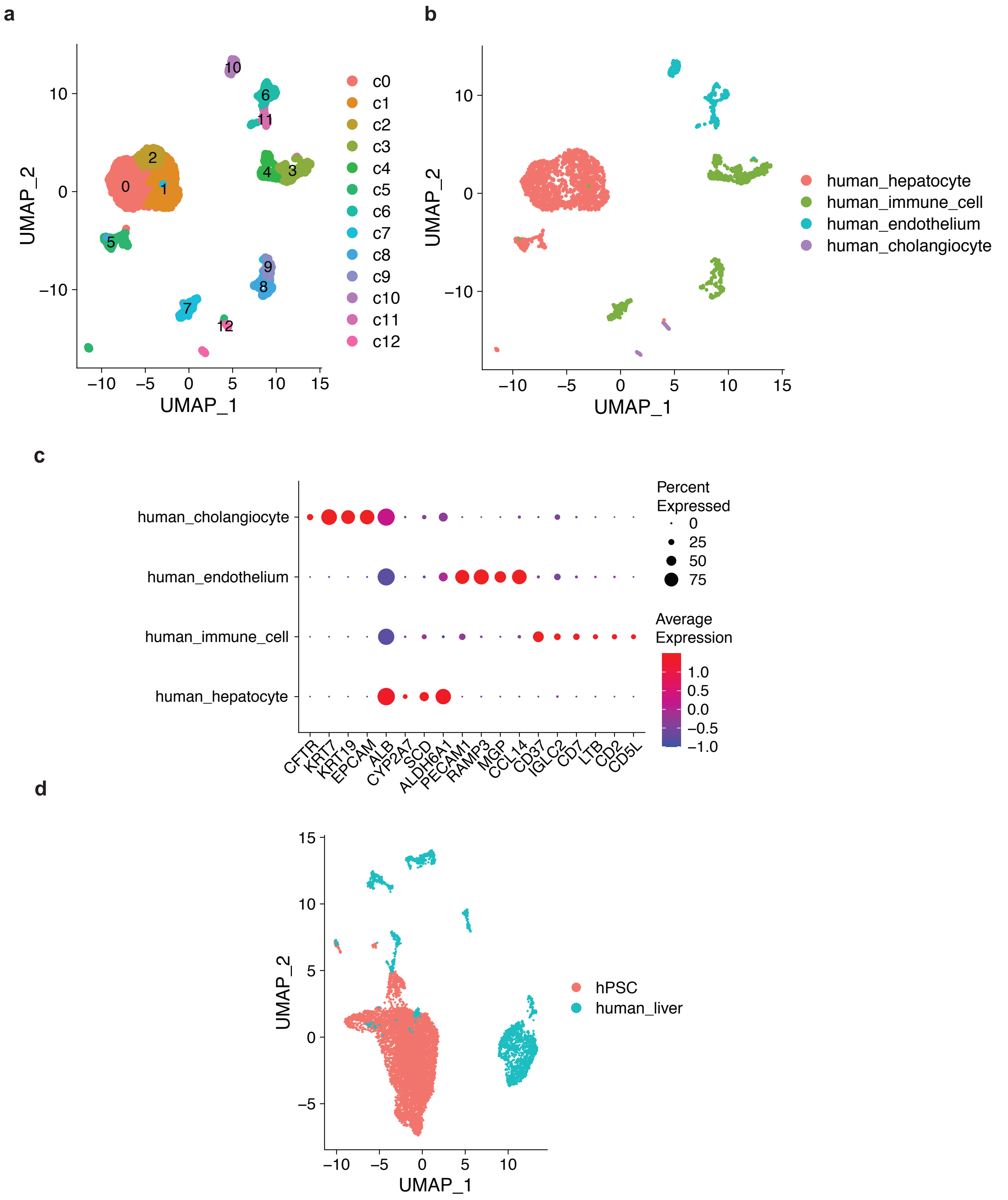

Supplementary Figure 16

Global gene analysis of human adult liver.

(a) UMAP projection of human adult liver cells labelled by cell cluster. (b) UMAP projection of human hepatocyte, immune cell, endothelium and cholangiocyte. (c) Dot plots displaying the representative core genes in the 4 main cell types in the human adult liver dataset. (d) UMAP projection of the integrated hPSC-derived cholangiocyte and human adult liver data.

Supplementary Tables

Supplementary Table 1. Cell cycle status in hPSC-derived cholangiocyte clusters.

ClusterID	G1	G2M	S
0	1444	24	401
1	1171	8	373
2	915	18	310
3	677	42	498
4	740	16	95
5	454	4	31
6	305	9	44
7	0	209	131
8	90	9	3
9	8	0	23
10	22	0	1
11	17	0	3

Supplementary Table 2. Quantification of cell distribution of monolayer and cyst population in 2D dominant, 3D dominant and combine clusters.

	**2D**	**3D**
**Chol_combine**	0.715580239695995	0.284419760304005
**Chol_3D_dominant**	0.293089430894309	0.706910569105691
**Chol_2D_dominant**	0.979321753515302	0.0206782464846981
**Chol_hepatic_progenitor**	0.0143149284253579	0.985685071574642
**Chol_proliferative_cell**	0.697058823529412	0.302941176470588
**GI secretory cell (c8)**	0.382352941176471	0.617647058823529
**GI secretory cell (c9)**	0	1
**GI secretory cell (c10)**	0.0869565217391304	0.91304347826087
**GI secretory cell (c11)**	0.5	0.5

Supplementary Table 3. Pearson correlation coefficient between hPSC-derived cholangiocyte clusters and human adult liver clusters (related to Figure 8h).

	**Chol_ combine**	**Chol_3D_ dominant**	**Chol_2D_ dominant**	**Chol_prolife rative_cell**	**GI secretory cell (c8)**	**GI secretory cell (c11)**	**Chol_hepatic_ progenitor**	**GI secretory cell (c10)**	**GI secretory cell (c9)**	**human_chol angiocyte**	**human_im mune_cell**	**human_he patocyte**	**human_en dothelium**
**Chol_combine**	1	0.86556759273305	0.937003606412429	0.906610728950812	0.431004859719989	0.383314695051695	0.547534321368245	0.0986434169503258	0.406689960931043	0.297603094180693	0.289593658647243	0.124249659707914	0.24315928624624
**Chol_3D_dominant**	0.86556759273305	1	0.79644178858359	0.868802133962525	0.449056226666486	0.531750913894766	0.745489112576346	0.140197552322878	0.565352267777755	0.44485775897689	0.467533633339467	0.199753653393421	0.375054242030586
**Chol_2D_dominant**	0.937003606412429	0.79644178858359	1	0.856940299113817	0.612232315623656	0.379325089047852	0.570098745616535	0.0886786471406535	0.438391318525312	0.26513480503985	0.296662106728521	0.106285572557229	0.268658003327273
**Chol_proliferative_cell**	0.906610728950812	0.868802133962525	0.856940299113817	1	0.443821636658391	0.414492212504687	0.588689206149809	0.100975677919983	0.459100385560331	0.34090116703361	0.291824589744359	0.138934138026821	0.251264529639942
**GI secretory cell (c8)**	0.431004859719989	0.449056226666486	0.612232315623656	0.443821636658391	1	0.279981215033299	0.446919738671561	0.0608116398217735	0.364025991323051	0.173397558116614	0.259076063199433	0.0596073116562302	0.333320282852697
**GI secretory cell (c11)**	0.383314695051695	0.531750913894766	0.379325089047852	0.414492212504687	0.279981215033299	1	0.495677717529169	0.0976109180764678	0.377622090867502	0.236782644686379	0.429492918334866	0.130026100026159	0.302385284628785
**Chol_hepatic_progenitor**	0.547534321368245	0.745489112576346	0.570098745616535	0.588689206149809	0.446919738671561	0.495677717529169	1	0.154905166796591	0.6060957050663	0.374434286222846	0.477263858278797	0.221323799677211	0.35432527070485
**GI secretory cell (c10)**	0.0986434169503258	0.140197552322878	0.0886786471406535	0.100975677919983	0.0608116398217735	0.0976109180764678	0.154905166796591	1	0.0767736422908112	0.0735540431667983	0.0792833346798614	0.100204526723651	0.060239667974537
**GI secretory cell (c9)**	0.406689960931043	0.565352267777755	0.438391318525312	0.459100385560331	0.364025991323051	0.377622090867502	0.60609570500663	0.0767736422908112	1	0.219836250585716	0.370769897974125	0.0827937191648274	0.283926712975513
**human_cholangiocyte**	0.297603094180693	0.44485775897689	0.26513480503985	0.34090116703361	0.173397558116614	0.236782644686379	0.374434286222846	0.0735540431667983	0.219836250585716	1	0.228162888010546	0.535191870324963	0.303297688992009
**human_immune_cell**	0.289593658647243	0.467533633339467	0.296662106728521	0.291824589744359	0.259076063199433	0.429492918334866	0.477263858278797	0.0792833346798614	0.370769897974125	0.228162888010546	1	0.1543646431444	0.440318454863807
**human_hepatocyte**	0.124249659707914	0.199753653393421	0.106285572557229	0.138934138026821	0.0596073116562302	0.130026100026159	0.221323799677211	0.100204526723651	0.0827937191648274	0.535191870324963	0.1543646431444	1	0.263909757481258
**human_endothelium**	0.24315928624624	0.375054242030586	0.268658003327273	0.251264529639942	0.333320282852697	0.302385284628785	0.35432527070485	0.060239667974537	0.283926712975513	0.303297688992009	0.440318454863807	0.263909757481258	1

Supplementary Table 4. Primary Antibodies used for Immunohistochemistry and Flow Cytometry analysis.

Antibody	Company	Product Codes	IgG Species	Conjugate	Dilution
CFTR (24-1)	R and D	MAB25031	Mouse	none	1:200
CFTR (13-1)	R and D	MAB1660	Mouse	none	1:200
CK7	Abcam	ab68459	Rabbit	none	1:200
Acethylated alpha tubulin	Sigma-Aldrich	T7451	Mouse	none	1:800
ZO-1	Themo Fisher	40-2200	Rabbit	none	1:400
ARL13b	Proteintech	17711 1-AP	Rabbit	none	1:600
human CK19	Abcam	ab52625	Rabbit	none	1:400
human CK19	DAKO	M0888	Mouse	none	1:20
mouse CK19	Abcam	133496	Rabbit	none	1:400
Mitochondria (113-1)	Millipore	MAB1273	Mouse	none	1:100
AFP	DAKO	A0008	Mouse	none	1:2000
SOX9	Abcam	ab76997	Mouse	none	1:400
ASBT (C14)	Santa Cruz	sc27493	Goat	none	1:50
SCTR	Sigma-Aldrich	HPA007269	Rabbit	none	1:50
SLC4A2	Sigma-Aldrich	HPA019339	Rabbit	none	1:50
TGR5	Invitrogen	PA5-27076	Rabbit	none	1:100
ALB	Bethyl	A80-129A	Goat	none	1:200
DHIC5-4D9	gift from oregon		Mouse (IgM)	none	1:20
CD117 (c-KIT)	BD pharmingen	BD340529	Mouse (IgG1)	PE	1:50
CD184 (CXCR4)	BD pharmingen	BD555976	Mouse (IgG1)	APC	1:50
CD326 (EPCAM)	eBioscience	12-9326-73	Mouse (IgG1)	PE	1:200
SSEA4	BD Horizon	561156	Mouse (IgG3)	V450	1:100
TRA-1-60	Biolegend	330605	Mouse (IgM)	Alexa Fluor 647	1:100
OCT3/4	BD pharmingen	560791	Mouse (IgG1)	Alexa Fluor 488	1:100
SOX2	BD pharmingen	561556	Mouse (IgG1)	PE	1:100
NANOG	BD pharmingen	561506	Mouse (IgG1)	PerCP-Cy5.5	1:100

Supplementary Table 5. Secondary Antibodies used for Immunohistochemistry and Flow Cytometry analysis.

Antibody	Company	Product Codes	Dilution
IgG Donkey anti-Mouse Alexa488	Invitrogen	A21202	1:400
IgG Donkey anti-Rabbit Alexa555	Invitrogen	A31572	1:400
IgG Donkey anti-Mouse Alexa555	Invitrogen	A31570	1:400
IgG Donkey anti-Rabbit Alexa488	Invitrogen	A21206	1:400
IgG Donkey anti-Goat Alexa488	Invitrogen	A11055	1:400
IgG Donkey anti-Goat Alexa647	Invitrogen	A21447	1:400
IgM Goat anti-Mouse APC	Jackson ImmunoResearch	115-136-075	1:200

Supplementary Table 6. Primers used for RT-PCR analysis.

Gene	Sequences (Forward)	Sequences (Reverse)
CFTR	5'-AGGACTATGGACACTTCGTGCCTT-3'	5'-ATTTGGAACCAGCGCAGTGTTGAC-3'
CK7	5'-AAGGATGCTCGTGCCAAG-3'	5'-AGCTTCACGCTCATGAGTTC-3'
SPP1	5'-CGAGGAGTTGAATGGTGCATA-3'	5'-TCCAGCTGACTCGTTTCATAAC-3'
TRPV4	5'-AGGTGAACTGGTCTCACTGG-3'	5'-GCGAGAAGCCATAATACTGGTAG-3'
PKD1	5'-GGACAAGGTGTGAGCCTGAG-3'	5'-AGCTGGTAGACGTCCTCTGT-3'
PKD2	5'-TTCCCAGATCAGTCATGGTTTAG-3'	5'-CCTTCCATGCCTTCTGTAGATT-3'
ALB	5'-GTGAAACACAAGCCCAAGGCAACA-3'	5'-TCAGCCTTGCAGCACTTCTCTACA-3'
AFP	5'-ACAGAGGAACAACTTGAGGCTGTC-3'	5'-AGCAAAGCAGACTTCCTGTTCCTG-3'
TMEM16A	5'-AAGTACTCGACGCTCCCGGCC-3'	5'-ATAAGGAGTTCAGCAGCGTGCCC-3'
AQP1	5'-TCTTCCGTGCCCTCATGTA-3'	5'-CAAGCGAGTTCCCAGTCAG-3'
SLC5A1	5'-TCAGGAGAGCCTATGACCTATT-3'	5'-GGTGTCCGTCATCTTCATCTT-3'
SLC4A2/AE2	5'-GGCATCTGTGCCCTCTTT-3'	5'-TCCTGAATCTTGGGCTTGTC-3'
ITPR3	5'-CGAGATGCTGCCCTTTGA-3'	5'-CAGAGACGGGCAAACTTGA-3'
P2YR	5'-GACTTCTTGTACGTGCTGACT-3'	5'-GCTGCCATAGAGGTTCACAT-3'
SOX9	5'-TGCATTTCCTCCTGCCTTTGCTTG-3'	5'-GGGCACTTATTGGCTGCTGAAACA-3'
SCTR	5'-TGCATCATGGCCAACTACTC-3'	5'-AATCCCTGGAGGTACTTTCTTTC-3'
TBP	5-'TGAGTTGCTCATACCGTGCTGCTA-3'	5'-CCCTCAAACCAACTTGTCAACAGC-3'

Supplementary Table 7. RNA control samples used for RT-PCR analysis.

RNA	Source	Sex	Lot	Company	Product number
human gall bladder	normal gall bladder from 34-years old	Female	A509245	BioChain	R1234118-10
human adult liver	normal livers pooled from 3 Asians (22-64-years old)	Male	1402003	Clonetech	636531
human fetal liver (Fig1b)	pooled from 63 spontaneously aborted fetus, aged 22-40 weeks	Male and Female	7030173	Clonetech	636540
human fetal liver (Fig2d,3e,Supp11a)	fetal liver from 24-weeks gestation	Male	1394	cell applications	1F21-50
human pancreas	normal pancreas from a 35-years old Caucasian	Male	1703157A	Clonetech	636577

Supplementary Tables 8. Authentication of new iPSC lines

WT01

Test Description	Method	Expected Result	Result
**Expression of pluripotency-associated proteins**	Flow cytometry	≥ 80% of population is positive for expression of surface markers (SSEA4, Tra-1-60), and intracellular markers (OCT4, SOX2).	**Antigen** **% Expressing-cells**<br>SSEA4$^+$/Tra-1-60$^+$ 96.8%<br>OCT4$^+$/SOX2$^+$ 99.5%<br>(Histograms shown in Figure 1A)
	Immunofluorescence	Positive fluorescence of surface markers (SSEA4, TRA-1-60) and intracellular markers (OCT4, NANOG)	Positive fluorescence signals obtained using all antibodies (Representative images in Figure 1B)
**Gene expression of pluripotency markers**	qRT-PCR	≥ 80% expression measured in hESC reference standard (HES2 hESCs on Matrigel).	**Gene** **Relative Expression**<br>OCT4 102%<br>NANOG 94%<br>DNMT3B 88%
**Germ layer differentiation**	Directed differentiation followed by qRT-PCR analysis	Increased expression of germ lineage-specific marker relative to starting pluripotent cell population	**Germ Layer** **Gene** **Fold Induction**<br>Endoderm SOX17 832<br>Mesoderm HAND1 12,708<br>Ectoderm SOX1 666
**Mycoplasma**	Lonza MycoAlert Plus kit	None detected	None detected
**Identity**	STR: PCR profiling of 9 STR regions plus Amelogenin for gender determination.	Consistent with expected[1]	Consistent with parental - Amel: XX<br>CSF1PO:10,12 D21S11:28,30 THOI:6,7<br>D13S317:12,12 D5S818:11,13 TPOX: 8,11<br>D16S539:11,12 D7S820:10,12 vWA:17,19
**Karyotype**	G-banding analysis detecting structural abnormality of size >3-10Mb	Normal karyotype, 46 XX or 46 XY 19/20 cells normal[2]	Normal karyotype, 46 XX at passage 5
**Post-Thaw Viability**	Cell count and viability using Nucleocounter	Viable cell count and viability 6 days post thaw	Viable cell count 1.23 E+06<br>Viability 90%
**Residual Sendai**	RT-PCR against Sendai viral elements	None detected in PCR amplification	None detected

CF01a

Test Description	Method	Expected Result	Result
**Expression of pluripotency-associated proteins**	Flow cytometry	≥ 80% of population is positive for expression of surface markers (SSEA4, Tra-1-60), and intracellular marker (OCT4).	**Antigen** **% Expressing-cells**<br>SSEA4 99.6%<br>Tra-1-60 99.7%<br>OCT4 99.3%<br>SOX2 99.7%<br>(Histograms shown in Figure 1)
**Gene expression of pluripotency markers**	qRT-PCR	≥ 80% expression measured in hESC reference standard (HES2 hESCs on Matrigel).	**Gene** **Relative Expression**<br>OCT4 96%<br>NANOG 99%<br>DNMT3B 89%
**Germ layer differentiation**	Directed Differentiation Followed by qPCR	Increased expression of germ lineage-specific marker relative to starting pluripotent cell population	**Germ Layer** **Gene** **Fold Induction**<br>Endoderm SOX17 9,500<br>Mesoderm HAND1 68,000<br>Ectoderm SOX1 362
**Definitive endoderm differentiation – gene expression**	Directed Differentiation Followed by qPCR	Increased expression of additional endoderm lineage-specific marker relative to starting pluripotent cell population	**Gene** **Fold Induction**<br>GATA 6 9,400<br>GATA 4 2,200<br>FOXA2 1,300
**Definitive endoderm differentiation – protein expression**	Directed Differentiation Followed by Flow cytometry	≥ 80% of population is double positive for expression of DE markers (cKIT and CXCR4)	**% Expressing-cells**<br>97.5%<br>(Histograms shown in Figure 2)
**Mycoplasma**	Lonza MycoAlert Plus kit	None detected	None detected
**Identity**	STR: PCR profiling of 9 STR regions plus Amelogenin for gender determination.	Consistent with expected[1]	Consistent with parental - Amel: XY<br>CSF1PO:12,12 D21S11:29,29 THOI:9.3,9.3<br>D13S317:11,12 D5S818:11,12 TPOX: 11,11<br>D16S539:11,12 D7S820:7,8 vWA:17,18
**Karyotype**	G-banding analysis detecting structural abnormality of size >3-10Mb	Normal karyotype, 46 XX or 46 XY 19/20 cells normal[2]	Normal karyotype, 46 XY at passage 3+5
**Post-Thaw Viability**	Cell count and viability using Nucleocounter	Viable cell count and viability within 7 days post thaw	Viable cell count 2.89E+05<br>Viability 83.50%
**Residual Sendai**	RT-PCR against Sendai viral elements	None detected in PCR amplification	None detected

Supplementary Tables 8. Authentication of new iPSC lines

CF01b

Test Description	Method	Expected Result	Result
**Expression of pluripotency-associated proteins**	Flow cytometry	≥ 80% of population is positive for expression of surface markers (SSEA4, Tra-1-60), and intracellular marker (OCT4).	**Antigen** **% Expressing-cells**<br>SSEA4 98.8%<br>Tra-1-60 99.2%<br>OCT4 98.8%<br>SOX2 99.3%<br>(Histograms shown in Figure 1)
**Gene expression of pluripotency markers**	qRT-PCR	≥ 80% expression measured in hESC reference standard (HES2 hESCs on Matrigel).	**Gene** **Relative Expression**<br>OCT4 146%<br>NANOG 96%<br>DNMT3B 114%
**Germ layer differentiation**	Directed Differentiation Followed by qPCR	Increased expression of germ lineage-specific marker relative to starting pluripotent cell population	**Germ Layer** **Gene** **Fold Induction**<br>Endoderm SOX17 3,300<br>Mesoderm HAND1 16,400<br>Ectoderm SOX1 181
**Definitive endoderm differentiation – gene expression**	Directed Differentiation Followed by qPCR	Increased expression of additional endoderm lineage-specific marker relative to starting pluripotent cell population	**Gene** **Fold Induction**<br>GATA 6 4,500<br>GATA 4 870<br>FOXA2 310
**Definitive endoderm differentiation – protein expression**	Directed Differentiation Followed by Flow cytometry	≥ 80% of population is double positive for expression of DE markers (cKIT and CXCR4)	**% Expressing-cells**<br>95.7%<br>(Histogram shown in Figure 1)
**Mycoplasma**	Lonza MycoAlert Plus kit	None detected	None detected
**Identity**	STR: PCR profiling of 9 STR regions plus Amelogenin for gender determination.	Consistent with expected[1]	Consistent with parental - Amel: XY<br>CSF1PO:12,12 D21S11:29,29 THOI:9.3,9.3<br>D13S317:11,12 D5S818:11,12 TPOX: 11,11<br>D16S539:11,12 D7S820:7,8 vWA:17,18
**Karyotype**	G-banding analysis detecting structural abnormality of size >3-10Mb	Normal karyotype, 46 XX or 46 XY 19/20 cells normal[2]	Normal karyotype, 46 XY at passage 3+5
**Post-Thaw Viability**	Cell count and viability using Nucleocounter	Viable cell count and viability within 7 days post thaw	Viable cell count 2.45E+06<br>Viability 93%
**Residual Sendai**	RT-PCR against Sendai viral elements	None detected in PCR amplification	None detected

CF01MC

Test Description	Method	Expected Result	Result
**Expression of pluripotency-associated proteins**	Flow cytometry	≥ 80% of population is positive for expression of surface markers (SSEA4, Tra-1-60), and intracellular marker (OCT4).	**Antigen** **% Expressing-cells**<br>SSEA4 99.5%<br>Tra-1-60 96.3%<br>OCT4 99.9%<br>SOX2 99.8%<br>(Histograms shown in Figure 1)
**Gene expression of pluripotency markers**	qRT-PCR	≥ 80% expression measured in hESC reference standard (HES2 hESCs on Matrigel).	**Gene** **Relative Expression**<br>OCT4 100%<br>NANOG 125%<br>DNMT3B 228%
**Mycoplasma**	Lonza MycoAlert Plus kit	None detected	None detected
**Identity**	STR: PCR profiling of 9 STR regions plus Amelogenin for gender determination.	Consistent with expected[1]	Consistent with parental - Amel: XY<br>CSF1PO: 12,12 D21S11:29,29 THOI:9.3,9.3<br>D13S317:11,12 D5S818:11,12 TPOX: 11,11<br>D16S539:11,12 D7S820:7,8 vWA:17,18
**Karyotype**	G-banding analysis detecting structural abnormality of size >3-10Mb	Normal karyotype, 46 XX or 46 XY 19/20 cells normal[2]	Normal karyotype, 46 XY at passage P3+23
**Post-Thaw Viability**	Cell count and viability using Nucleocounter	Viable cell count and viability within 7 days post thaw	Viable cell count 5.20E6<br>Viability 80.1%
**Sequence confirmation**	PCR and Sanger sequencing of targeted locus	Desired mutation	Full sequence of wild-type and targeted allele show no aberrant mutations (Figure 2)
**Off-target analysis**	PCR and Sanger sequencing of genomic sites likely to be subject to off-target cleavage	No mutations in putative off-target cut sites	Sequenced 5 genomic sites most likely to be inappropriately cut by designed gRNA and found no aberrant mutations (Table 1)
**Clonal Population**	TOPO cloning of PCR from genomic DNA and sequencing	Homozygous– 100% of individual TOPO reactions represent each allele	12 TOPO clones sequenced – all corrected

Supplementary Tables 8. Authentication of new iPSC lines

CF02a

Test Description	Method	Expected Result	Result
**Expression of pluripotency-associated proteins**	Flow cytometry	≥ 80% of population is positive for expression of surface markers (SSEA4, Tra-1-60), and intracellular marker (OCT4).	**Antigen** **% Expressing-cells**<br>SSEA4 98.4%<br>Tra-1-60 99.7%<br>OCT4 99.0%<br>SOX2 99.6%<br>(Histograms shown in Figure 1)
**Gene expression of pluripotency markers**	qRT-PCR	≥ 80% expression measured in hESC reference standard (HES2 hESCs on Matrigel).	**Gene** **Relative Expression**<br>OCT4 100%<br>NANOG 106%<br>DNMT3B 85%
**Germ layer differentiation**	Directed Differentiation Followed by qPCR	Increased expression of germ lineage-specific marker relative to starting pluripotent cell population	**Germ Layer** **Gene** **Fold Induction**<br>Endoderm SOX17 6,200<br>Mesoderm HAND1 38,700<br>Ectoderm SOX1 106
**Definitive endoderm differentiation – gene expression**	Directed Differentiation Followed by qPCR	Increased expression of additional endoderm lineage-specific marker relative to starting pluripotent cell population	**Gene** **Fold Induction**<br>GATA 6 7000<br>GATA 4 5,900<br>FOXA2 680
**Definitive endoderm differentiation – protein expression**	Directed Differentiation Followed by Flow cytometry	≥ 80% of population is double positive for expression of DE markers (cKIT and CXCR4)	**% Expressing-cells**<br>99.9%<br>(Histogram shown in Figure 1)
**Mycoplasma**	Lonza MycoAlert Plus kit	None detected	None detected
**Identity**	STR: PCR profiling of 9 STR regions plus Amelogenin for gender determination.	Consistent with expected[1]	Consistent with parental - Amel: XX<br>CSF1PO:10,12 D21S11:27,30 THOI:6,8<br>D13S317:8,14 D5S818:11,13 TPOX: 8,11<br>D16S539:8,13 D7S820:11,12 vWA:16,19
**Karyotype**	G-banding analysis detecting structural abnormality of size >3-10Mb	Normal karyotype, 46 XX or 46 XY 19/20 cells normal[2]	Normal karyotype, 46 XX at passage 3+5
**Post-Thaw Viability**	Cell count and viability using Nucleocounter	Viable cell count and viability within 7 days post thaw	Viable cell count 4.05E+06<br>Viability 90.3%
**Residual Sendai**	RT-PCR against Sendai viral elements	None detected in PCR amplification	None detected

CF02b

Test Description	Method	Expected Result	Result
**Expression of pluripotency-associated proteins**	Flow cytometry	≥ 80% of population is positive for expression of surface markers (SSEA4, Tra-1-60), and intracellular marker (OCT4).	**Antigen** **% Expressing-cells**<br>SSEA4 99.6%<br>Tra-1-60 99.7%<br>OCT4 99.3%<br>SOX2 99.7%<br>(Histograms shown in Figure 1)
**Gene expression of pluripotency markers**	qRT-PCR	≥ 80% expression measured in hESC reference standard (HES2 hESCs on Matrigel).	**Gene** **Relative Expression**<br>OCT4 153%<br>NANOG 151%<br>DNMT3B 201%
**Germ layer differentiation**	Directed Differentiation Followed by qPCR	Increased expression of germ lineage-specific marker relative to starting pluripotent cell population	**Germ Layer** **Gene** **Fold Induction**<br>Endoderm SOX17 4,000<br>Mesoderm HAND1 11,300<br>Ectoderm SOX1 200
**Definitive endoderm differentiation – gene expression**	Directed Differentiation Followed by qPCR	Increased expression of additional endoderm lineage-specific marker relative to starting pluripotent cell population	**Gene** **Fold Induction**<br>GATA 6 4,200<br>GATA 4 1,000<br>FOXA2 160
**Definitive endoderm differentiation – protein expression**	Directed Differentiation Followed by Flow cytometry	≥ 80% of population is double positive for expression of DE markers (cKIT and CXCR4)	**% Expressing-cells**<br>99.7%<br>(Histogram shown in Figure 1)
**Mycoplasma**	Lonza MycoAlert Plus kit	None detected	None detected
**Identity**	STR: PCR profiling of 9 STR regions plus Amelogenin for gender determination.	Consistent with expected[1]	Consistent with parental - Amel: XX<br>CSF1PO:10,12 D21S11:27,30 THOI:6,8<br>D13S317:8,14 D5S818:11,13 TPOX: 8,11<br>D16S539:8,13 D7S820:11,12 vWA:16,19
**Karyotype**	G-banding analysis detecting structural abnormality of size >3-10Mb	Normal karyotype, 46 XX or 46 XY 19/20 cells normal[2]	Normal karyotype, 46 XX at passage 3+5
**Post-Thaw Viability**	Cell count and viability using Nucleocounter	Viable cell count and viability within 7 days post thaw	Viable cell count 5.02E+05<br>Viability 88.7%
**Residual Sendai**	RT-PCR against Sendai viral elements	None detected in PCR amplification	None detected

Supplementary Tables 8. Authentication of new iPSC lines

CF02MC

Test Description	Method	Expected Result	Result
**Expression of pluripotency-associated proteins**	Flow cytometry	≥ 80% of population is positive for expression of surface markers (SSEA4, Tra-1-60), and intracellular marker (OCT4).	**Antigen** / **% Expressing-cells**<br>SSEA4 94.1%<br>Tra-1-60 87.4%<br>OCT4 99.9%<br>SOX2 99.7%<br>(Histograms shown in Figure 1)
**Gene expression of pluripotency markers**	qRT-PCR	≥ 80% expression measured in hESC reference standard (HES2 hESCs on Matrigel).	**Gene** / **Relative Expression**<br>OCT4 134%<br>NANOG 177%<br>DNMT3B 141%
**Mycoplasma**	Lonza MycoAlert Plus kit	None detected	None detected
**Identity**	STR: PCR profiling of 9 STR regions plus Amelogenin for gender determination.	Consistent with expected[1]	Consistent with parental - Amel: XX<br>CSF1PO:10,12 D21S11:27,30 THOI:6,8<br>D13S317:8,14 D5S818:11,13 TPOX: 8,11<br>D16S539:8,13 D7S820:11,12 vWA:16,19
**Karyotype**	G-banding analysis detecting structural abnormality of size >3-10Mb	Normal karyotype, 46 XX or 46 XY 19/20 cells normal[2]	Normal karyotype, 46 XX at passage P3+34
**Post-Thaw Viability**	Cell count and viability using Nucleocounter	Viable cell count and viability within 7 days post thaw	Viable cell count 2.94E6<br>Viability 90.3%
**Sequence confirmation**	PCR and Sanger sequencing of targeted locus	Desired mutation	Full sequence of wild-type and targeted allele show no aberrant mutations (Figure 2)
**Off-target analysis**	PCR and Sanger sequencing of genomic sites likely to be subject to off-target cleavage	No mutations in putative off-target cut sites	Sequenced 5 genomic sites most likely to be inappropriately cut by designed gRNA and found no aberrant mutations (Table 1)
**Clonal Population**	TIDER analysis of sequencing trace	>80% HDR efficiency indicates homozygous, single cell clonality	96.0% HDR efficiency (Figure 2C)

iPSC lines	Authentication Date
WT01	2015-09-28
CF01a	2017-03-03
CF01b	2017-03-03
CF01MC	2018-02-14
CF02a	2017-03-03
CF02b	2017-03-03
CF02MC	2019-05-10

Supplementary Figure 12a MC Uncropped western blots

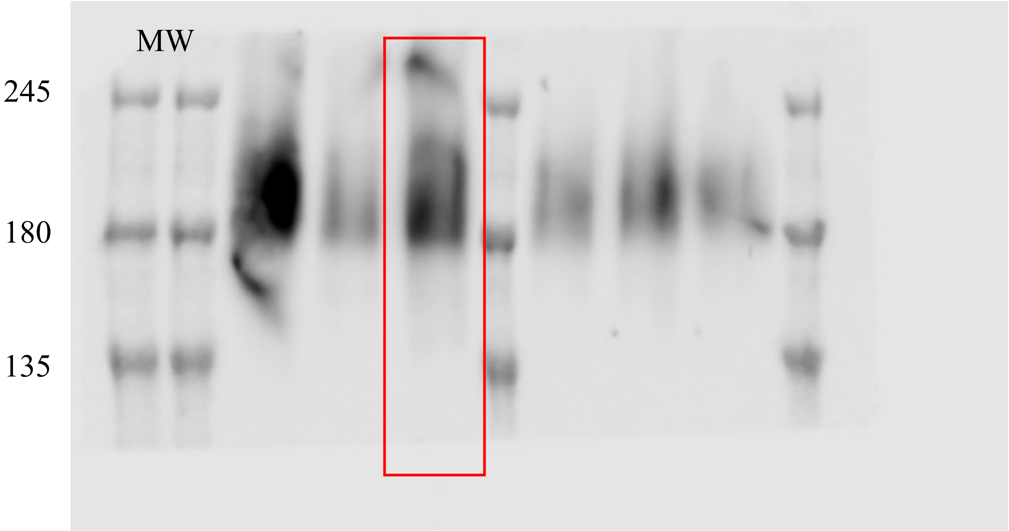

Wt CFTR

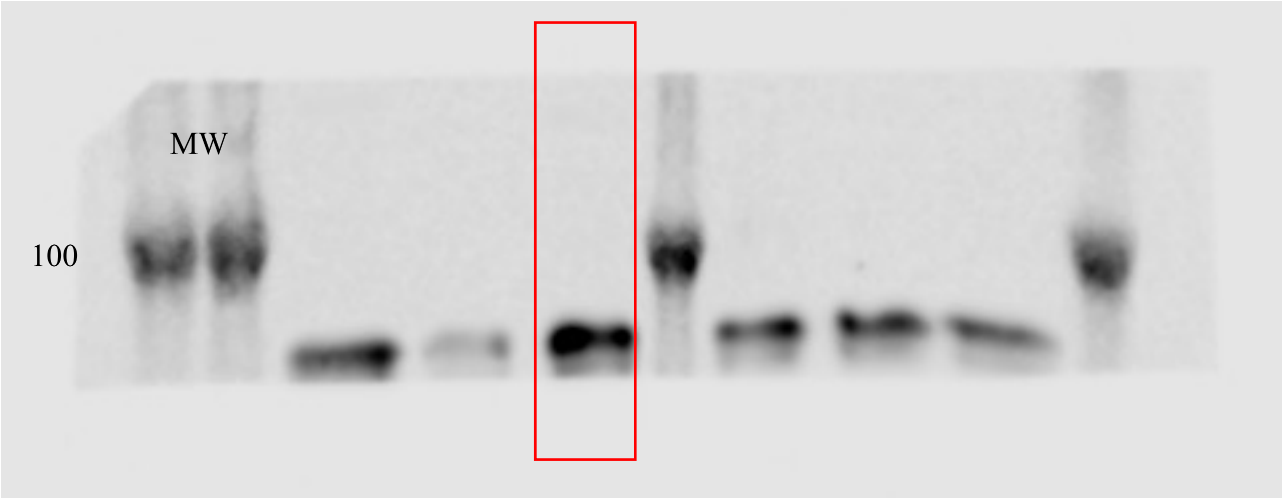

Calnexin

Supplementary Figure 12a CF Uncropped western blots

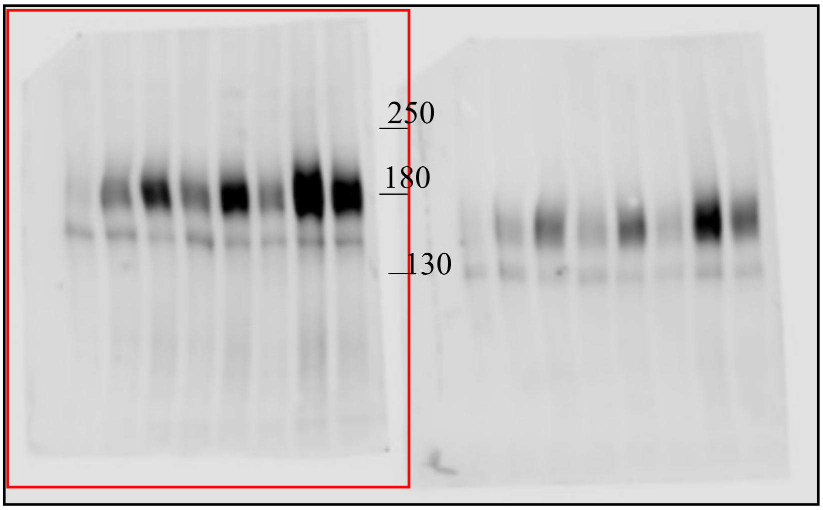

F508del CFTR

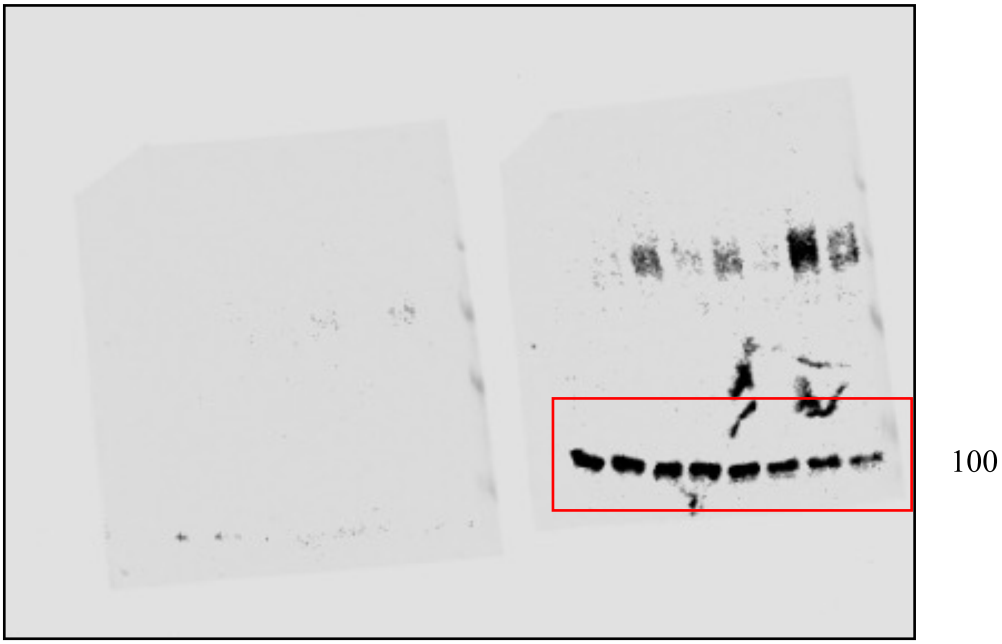

Calnexin

Supplementary Figure 12c MC Uncropped western blots

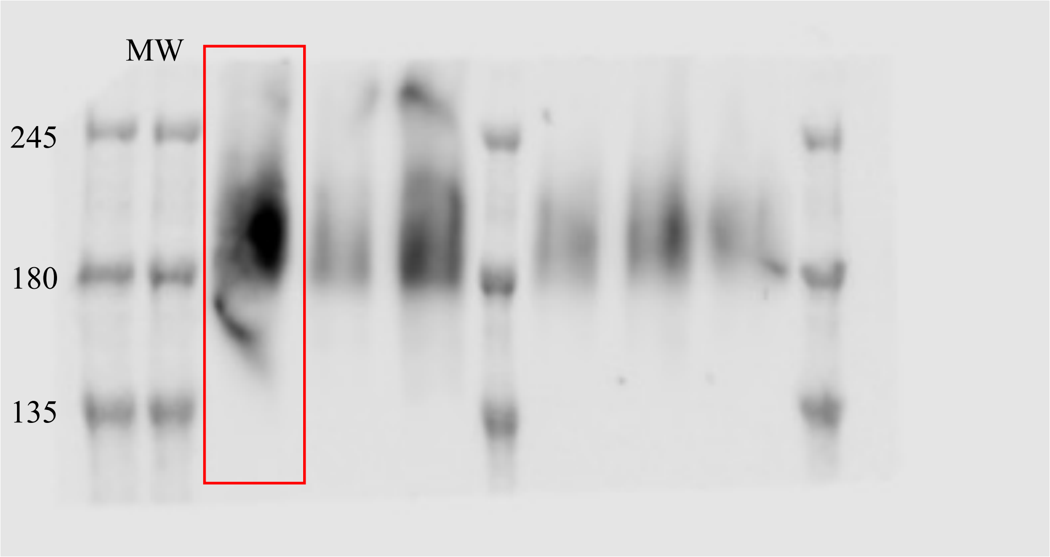

CFTR

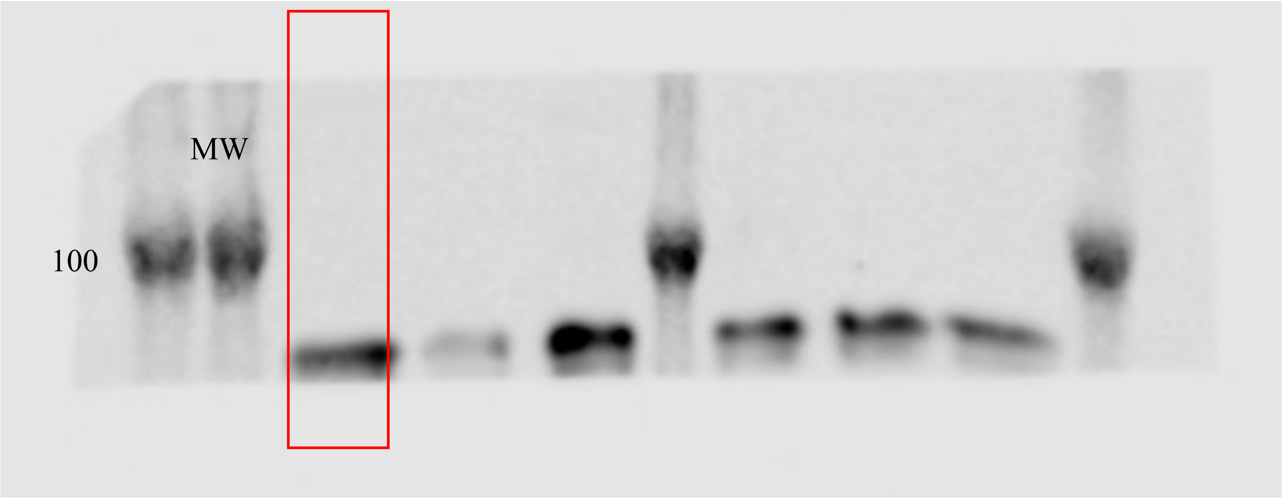

Calnexin

Supplementary Figure 12c CF Uncropped western blots

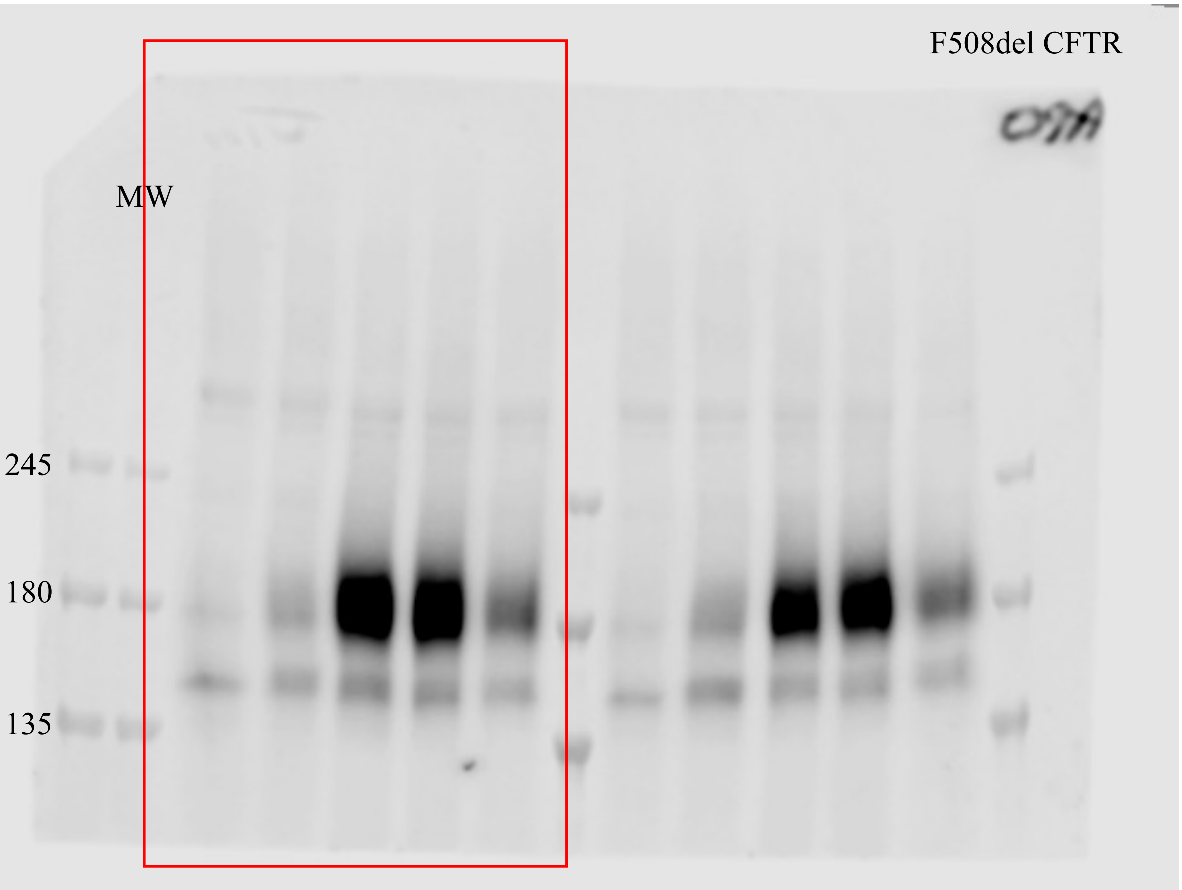

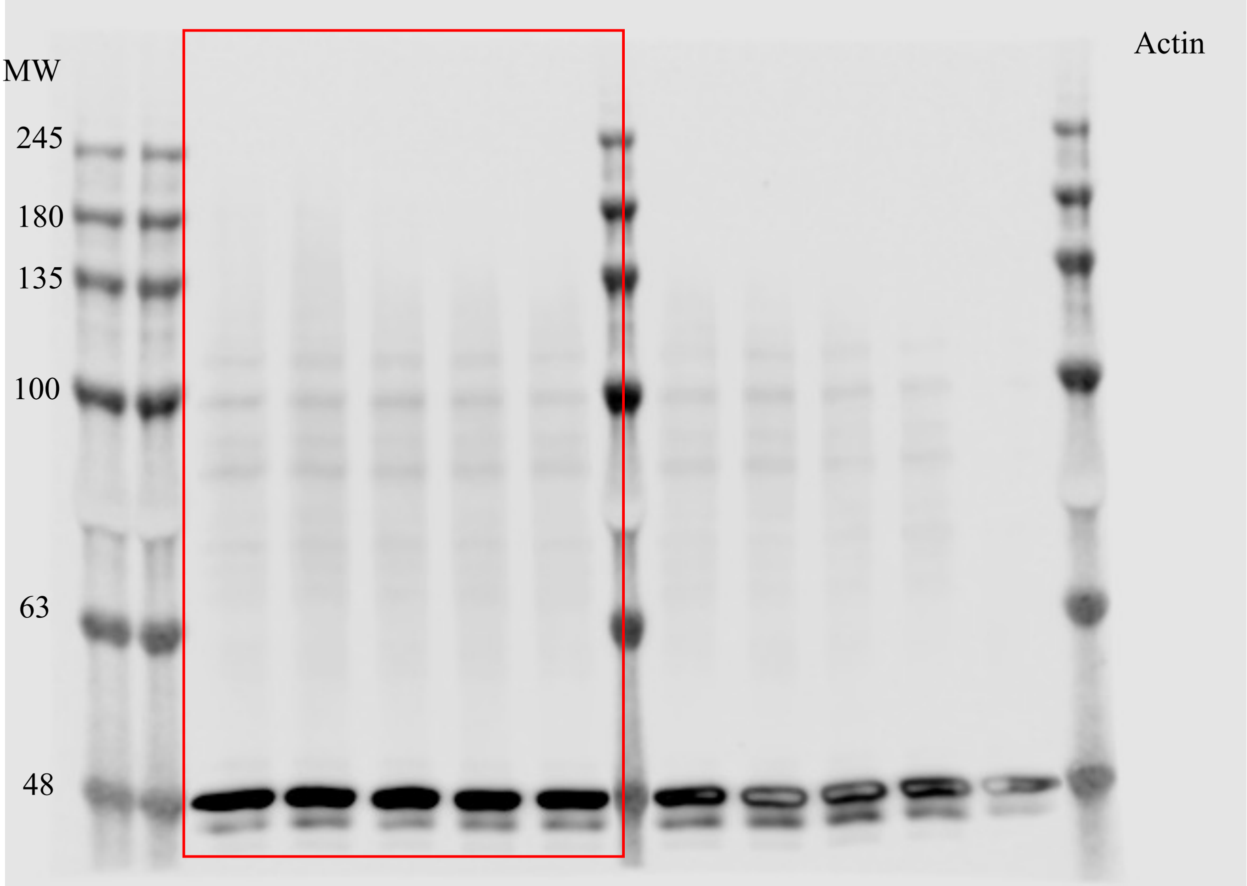

Supplementary Figure 12e CF Uncropped western blots

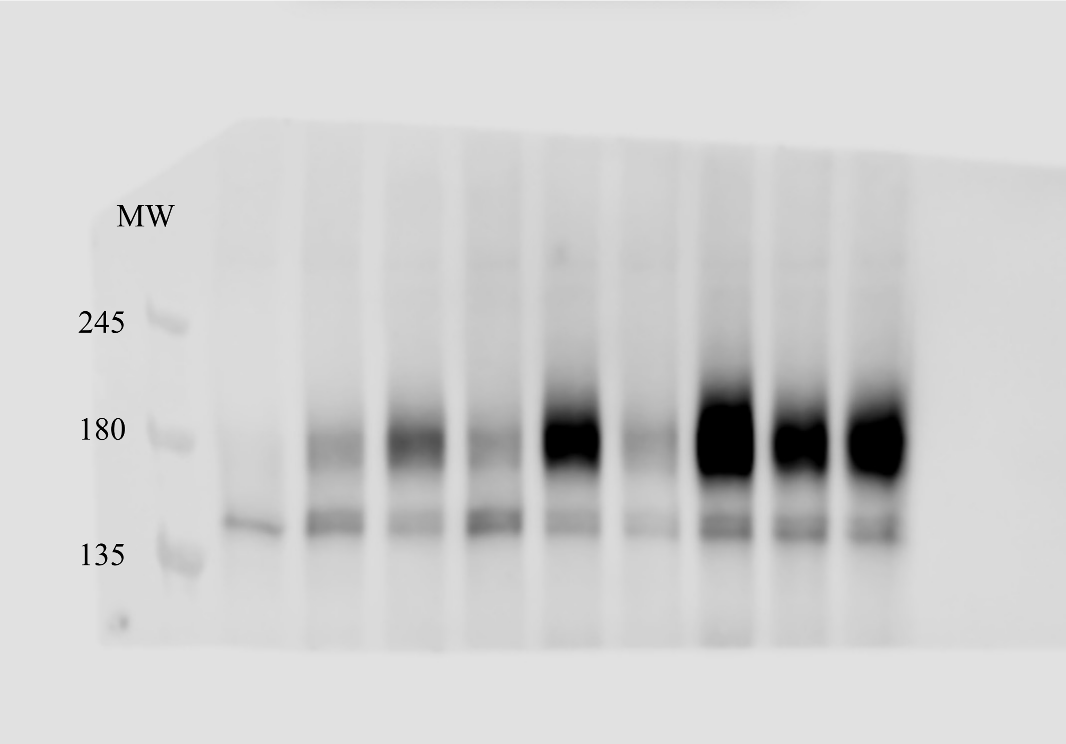

F508del CFTR

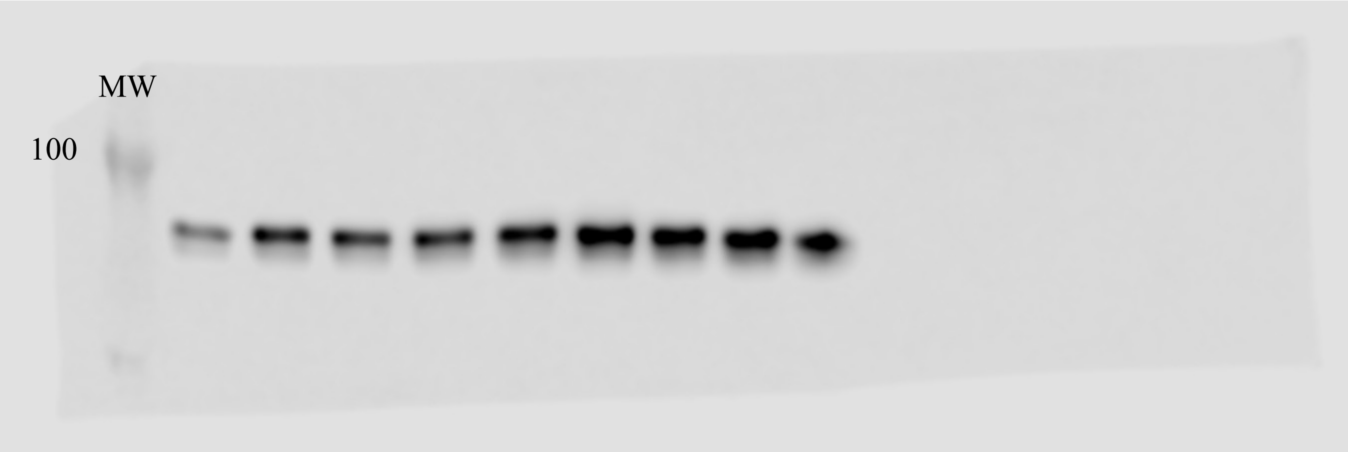

Actin

Supplementary Figure 12g MC Uncropped western blots

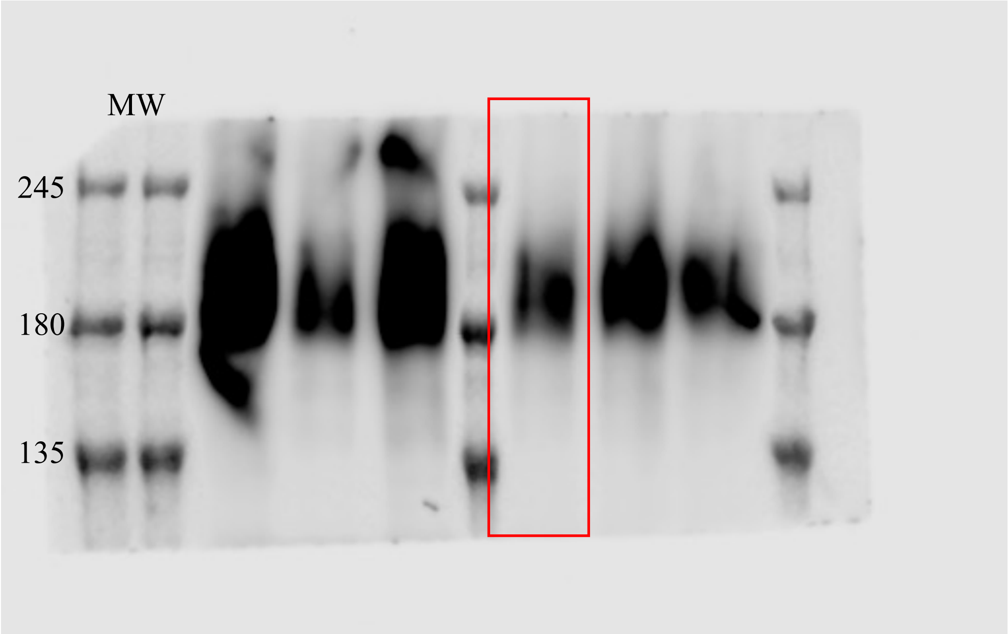

CFTR

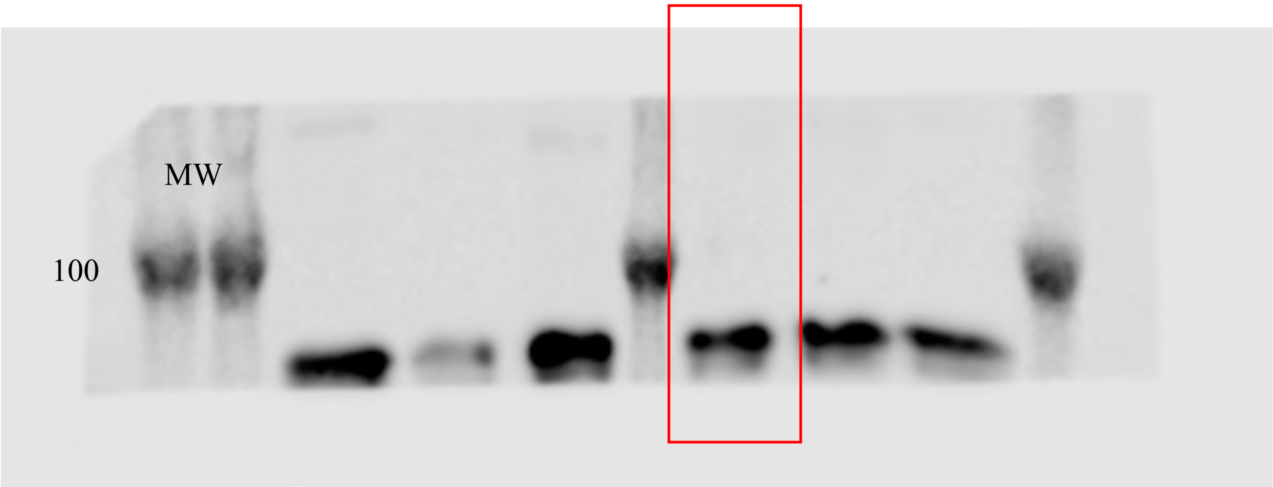

Calnexin

Supplementary Figure 12g CF Uncropped western blots

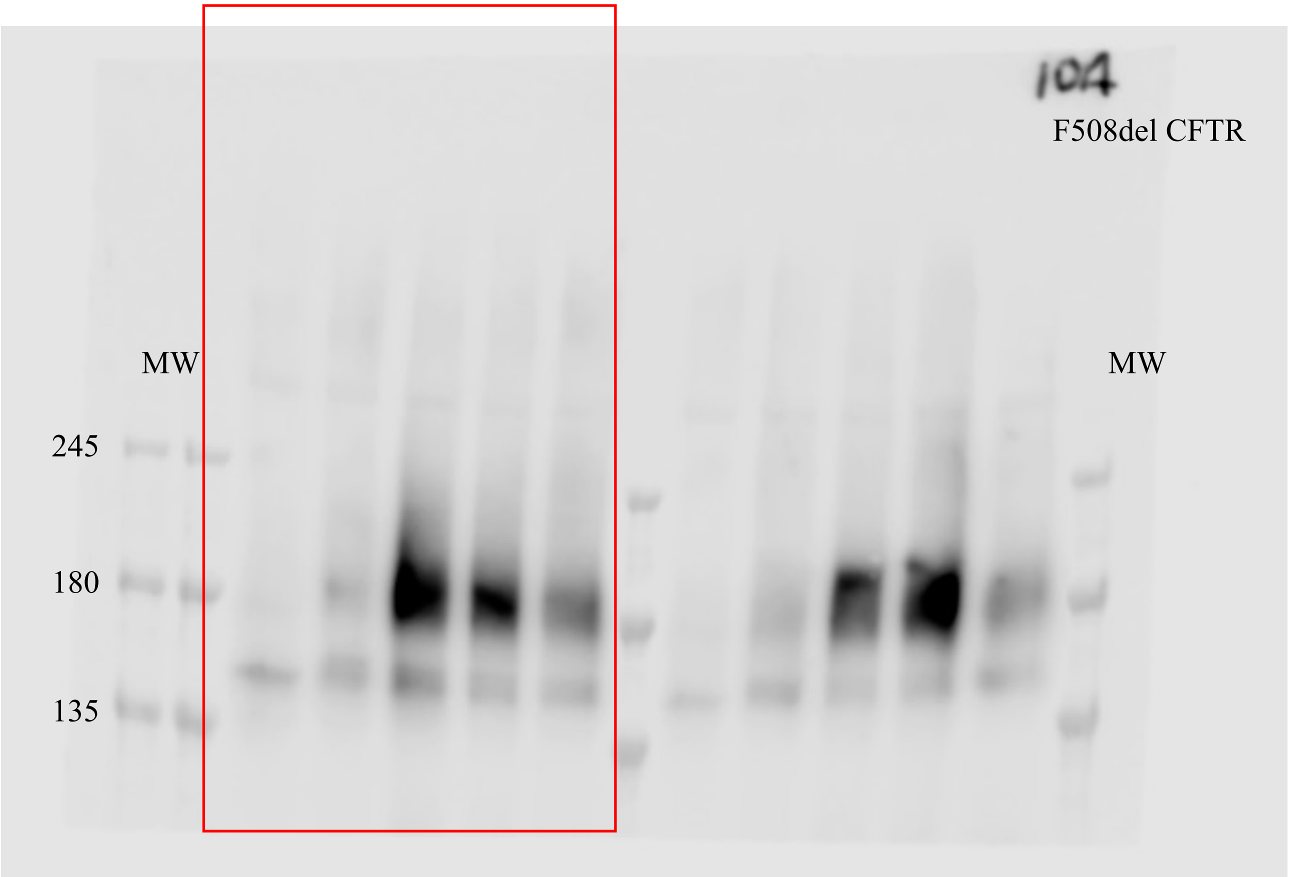

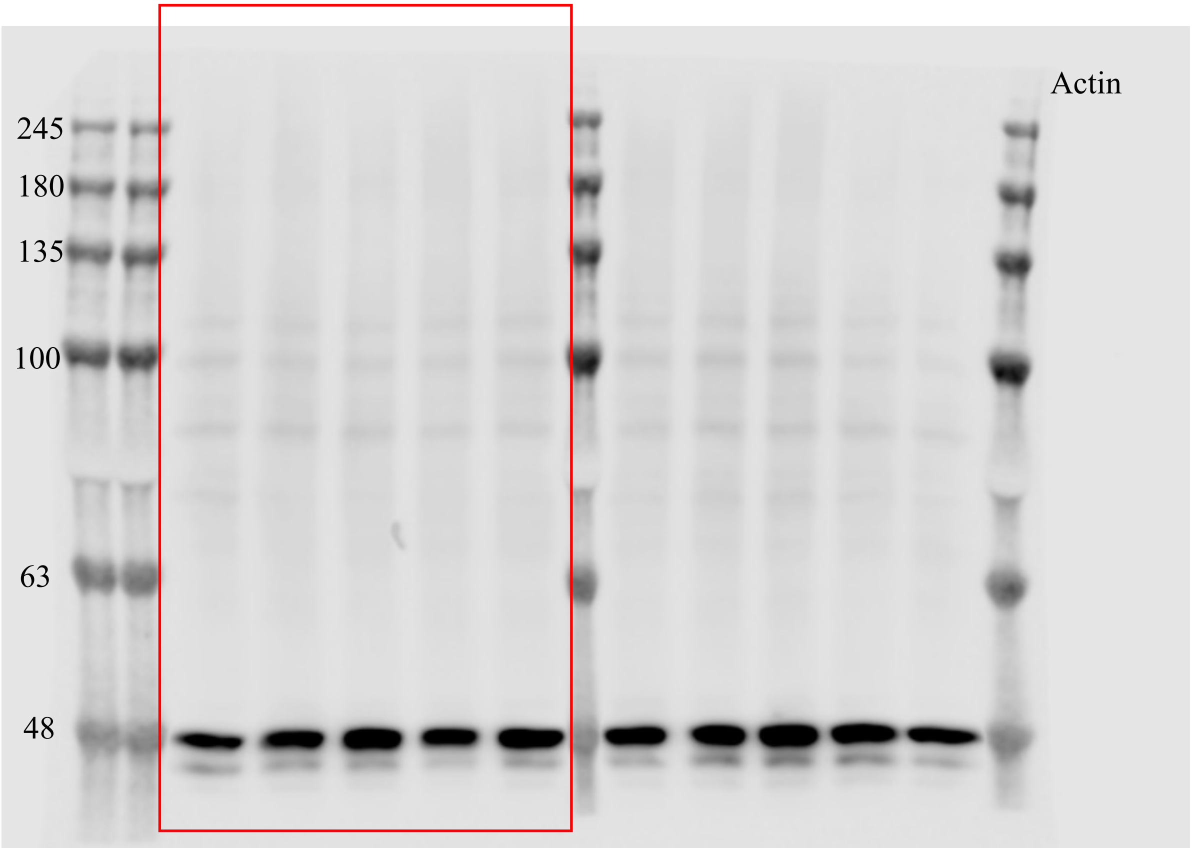

Supplement: Supplementary file 1 — Supplementary Information [file 41467_2021_26764_MOESM1_ESM.pdf]
