# Supplementary material for: Generation of functional ciliated cholangiocytes from human pluripotent stem cells
Source: Nat Commun. 2021 Nov 11;12:6504. doi: 10.1038/s41467-021-26764-0 (PMC8586142; doi:10.1038/s41467-021-26764-0)
Supplement: Supplementary file 3 — Reporting Summary [file 41467_2021_26764_MOESM3_ESM.pdf]

nature research

Corresponding author(s): Shinichiro Ogawa

Last updated by author(s): Oct 19, 2021

Reporting Summary

Nature Research wishes to improve the reproducibility of the work that we publish. This form provides structure for consistency and transparency in reporting. For further information on Nature Research policies, see our Editorial Policies and the Editorial Policy Checklist.

Statistics

For all statistical analyses, confirm that the following items are present in the figure legend, table legend, main text, or Methods section.

n/a	Confirmed	
[ ]	[x]	The exact sample size (n) for each experimental group/condition, given as a discrete number and unit of measurement
[ ]	[x]	A statement on whether measurements were taken from distinct samples or whether the same sample was measured repeatedly
[ ]	[x]	The statistical test(s) used AND whether they are one- or two-sided<br>*Only common tests should be described solely by name; describe more complex techniques in the Methods section.*
[x]	[ ]	A description of all covariates tested
[x]	[ ]	A description of any assumptions or corrections, such as tests of normality and adjustment for multiple comparisons
[ ]	[x]	A full description of the statistical parameters including central tendency (e.g. means) or other basic estimates (e.g. regression coefficient) AND variation (e.g. standard deviation) or associated estimates of uncertainty (e.g. confidence intervals)
[ ]	[x]	For null hypothesis testing, the test statistic (e.g. F, t, r) with confidence intervals, effect sizes, degrees of freedom and P value noted<br>*Give P values as exact values whenever suitable.*
[x]	[ ]	For Bayesian analysis, information on the choice of priors and Markov chain Monte Carlo settings
[x]	[ ]	For hierarchical and complex designs, identification of the appropriate level for tests and full reporting of outcomes
[ ]	[x]	Estimates of effect sizes (e.g. Cohen's d, Pearson's r), indicating how they were calculated

Our web collection on statistics for biologists contains articles on many of the points above.

Software and code

Policy information about availability of computer code

Data collection	BD LSRFortessa, GraphPad PRISM 8, ZEISS ZEN2.3, Leica CTR6000, NIKON A1R, FLIPR Tetra, Volocity 6.3, Spectra Max i3, Li-Cor Odyssey Fc, Leica SP8/STED, C1000 Touch Thermal Cycler, Hitachi SU3500 SEM, 10X Genomics Single Cell 3'v3, Chromium Single Cell Software Suite v3.1
Data analysis	FlowJo 10, GraphPad PRISM 8, ZEISS ZEN2.3, Leica Application Suite 3.8, NIKON Elements 5.20.02, Image Studio Lite5.2.5, ImageJ 1.51, Volocity 6.3, Imaris 9.5.1, R 3.61, Clusterprofiler 3.14.3, Seurat 3.2.2, Batchelor 1.2.4, Bioconductor 3.10, Corrplot 0.84 SeuratWrappers 0.3.0, msigdbr 7.2.1, org.Hs.eg.db 3.10.0

For manuscripts utilizing custom algorithms or software that are central to the research but not yet described in published literature, software must be made available to editors and reviewers. We strongly encourage code deposition in a community repository (e.g. GitHub). See the Nature Research guidelines for submitting code & software for further information.

Data

Policy information about availability of data

All manuscripts must include a data availability statement. This statement should provide the following information, where applicable:
- Accession codes, unique identifiers, or web links for publicly available datasets
- A list of figures that have associated raw data
- A description of any restrictions on data availability

The data supporting the findings of this study are available within the Article and its Supplementary Information files, and from the corresponding author on reasonable request. A Reporting Summary for this Article is available as a Supplementary Information file.
Raw scRNA seq data generated in this study has been deposited at the GEO database under accession code: GSE175502, referenced scRNA seq data of human adult liver is available under accession code: GSE115469. https://www.ncbi.nlm.nih.gov/geo/query/acc.cgi?

Field-specific reporting

Please select the one below that is the best fit for your research. If you are not sure, read the appropriate sections before making your selection.

- [x] Life sciences
- [ ] Behavioural & social sciences
- [ ] Ecological, evolutionary & environmental sciences

For a reference copy of the document with all sections, see nature.com/documents/nr-reporting-summary-flat.pdf

Life sciences study design

All studies must disclose on these points even when the disclosure is negative.

Sample size	No statistical analysis was used to predetermine sample size. Sample size was determined based on previous studies on the same subject. Sample size was at least n=3 biological replicates for all experiments.
Data exclusions	Data has only been excluded in FLIPR assay in case datasets were affected by machine error and over saturation of signal as they are not representative of a biological response.
Replication	Multiple cell lines have been examined and experiments were repeated successfully a minimum of three times.
Randomization	There was no randomization for the experiments as it did not involve clinical studies.
Blinding	The design of experiments did not require blinding as the outcome was not dependent on the judgment of the investigators and could not be influenced by prior knowledge of the groups.

Reporting for specific materials, systems and methods

We require information from authors about some types of materials, experimental systems and methods used in many studies. Here, indicate whether each material, system or method listed is relevant to your study. If you are not sure if a list item applies to your research, read the appropriate section before selecting a response.

Materials & experimental systems

n/a	Involved in the study	
[ ]	[x]	Antibodies
[ ]	[x]	Eukaryotic cell lines
[x]	[ ]	Palaeontology and archaeology
[ ]	[x]	Animals and other organisms
[x]	[ ]	Human research participants
[x]	[ ]	Clinical data
[x]	[ ]	Dual use research of concern

Methods

n/a	Involved in the study	
[x]	[ ]	ChIP-seq
[ ]	[x]	Flow cytometry
[x]	[ ]	MRI-based neuroimaging

Antibodies

Antibodies used

Primary Antibodies used in this study:
mouse anti-CFTR (24-1) (R and D; MAB25031) 1/200
mouse anti-CFTR (13-1) (R and D; MAB1660) 1/200
rabbit anti-CK7 (Abcam; ab68459) 1/200
mouse anti-acetylated alpha tubulin (Sigma-Aldrich; T7451) 1/800
rabbit anti-ZO-1 (Thermo Fisher; 40-2200) 1/400
rabbit anti-ARL13b (Proteintech;17711 1-AP) 1/600
rabbit anti-CK19 (Abcam; ab52625) 1/400
mouse anti-CK19 (DAKO; M0888) 1/20
rabbit anti-mouse-CK19 (Abcam; ab133496) 1/400
mouse anti-mitochondria (113-1) (Millipore; MAB1273) 1/100
mouse anti-AFP (DAKO; A0008) 1/2000
mouse anti-SOX9 (Abcam; ab76997) 1/400
goat anti-ASBT (C14) (Santa Cruz; sc27493) 1/50
rabbit anti-SCTR (Sigma; HPA007269) 1/50
rabbit anti-SLC4A2 (Sigma; HPA019339) 1/50
rabbit anti-TGR5 (Invitrogen; PA5-270765) 1/100
goat anti-ALB (Bethyl; A80-129A) 1/200
mouse anti-DHIC5-4D9 (gift from Oregon Stem cell Institute) 1/20, The antibody is also commercially available from Sigma (MABS2040)

mouse anti-CD117 (BD pharmingen; BD340529) 1/50
mouse anti-CD184 (BD pharmingen; BD555976) 1/50
mouse anti-CD326 (eBioscience; 12-9326-73) 1/200
mouse anti-SSEA4 (BD Horizon; 561156) 1/100
mouse anti-TRA-1-60 (Biolegend; 330605)1/100
mouse anti-OCT3/4 (BD pharmingen; 560791) 1/100
mouse anti-SOX2 (BD pharmingen; 561556) 1/100
mouse anti- NANOG (BD pharmingen; 561506) 1/100
mouse anti-alpha-actin (Sigma: A5044) 1/5000 WB
rabbit anti-calnexin (Sigma: C4731) 1/5000 WB

Secondary Antibodies:
IgG donkey anti-mouse Alexa488 (Invitrogen; A21202) 1/400
IgG donkey anti-rabbit Alexa555 (Invitrogen; A31572) 1/400
IgG donkey anti-mouse Alexa555 (Invitrogen; A31570) 1/400
IgG donkey anti-rabbit Alexa488 (Invitrogen; A21206) 1/400
IgG donkey anti-goat Alexa488 (Invitrogen; A11055) 1/400
IgG donkey anti-goat Alexa647 (Invitrogen; A21447) 1/400
IgM goat anti-mouse APC (Jackson ImmunoResearch; 115-136-075) 1/200

Validation

All commercial antibodies were validated by both manufactures and independent laboratories. Additional validation was performed by using negative controls and control tissue samples. The antibodies of DHIC5-4D9 was obtained from Oregon Stem Cell Institute and validated described in our previous publications (Dorrell et al. Stem Cell Res. 2008 Sep;1(3):183-94. doi: 10.1016/j.scr.2008.04.001, Galivo et al. Stem Cell Res. 2015 Jul; 15(1): 172–181. doi: 10.1016/j.scr.2015.06.004, Ogawa et al. Nat. Biotechnol. 2015 Aug;33(8):853-61. doi: 10.1038/nbt.3294).

Eukaryotic cell lines

Policy information about cell lines

Cell line source(s)

OP9 jagged1 was obtained form Dr. Juan Carlos Zúñiga-Pflücker (Sunnybrook Health Sciences Centre, Toronto). GCaMP3 cell line was obtained from Dr. Michael Laflamme (McEwen Stem Cell Institute, Toronto). iPSC-lines were obtained through the CFIT Program (https://lab.research.sickkids.ca/CFIT, SIckKids Research Ethics Board, Approval #44783) at SickKids in Toronto. H9 ES cell is commercially available and purchased from WiCell (Madison WI, USA).

Authentication

All cell lines were authenticated by providers. H9 and GCaMP3 were also authenticated in the following reference (H9: Ogawa et al. Nature Biotechnology 2015 doi: 10.1038/nbt.3294. GCaMP3: Shiba et al. Nature 2012 doi: 10.1038/nature11317, Zhu et al. Methods Mol Biol. 2011 doi: 10.1007/978-1-4939-1047-2_20). The lines generated at CCRM (Centre for Commercialization of Regenerative Medicine, Toronto) were authenticated by STR (Identity), G-banding (Karyotype), PCR and Sanger sequencing for targeted locus and off-target analysis. Data and Authentication Information of these iPSC-lines were shown in Supplementary Information.

Mycoplasma contamination

All cell lines tested negative for mycoplasma.

Commonly misidentified lines
(See ICLAC register)

No commonly misidentified cells were used.

Animals and other organisms

Policy information about studies involving animals; ARRIVE guidelines recommended for reporting animal research

Laboratory animals

The animals used for injury modeling in this study were TK-NOG mice (Hasegawa et al. Biochem Biophys Res Commun 2011 doi:10.1016/j.bbrc.2011.01.042, Higuchi et al. Hepatol Res. 2016 doi: 10.1111/hepr.12644). Ganciclovir (GCV; 10 mg/kg) was administered into adult 7-8 week-old male TK-NOG mice 7 to 10 days prior to the transplantation inducing significant liver damage. The level of serum ALT activity in TK-NOG mice was closely monitored. The NSG mouse line was purchased from the Jackson Laboratory (Bar Harbor, ME) and 8-10 week-old male NSG mice were used for transplantation. Day 55 monolayer cholangiocytes were dissociated by trypsin-EDTA and one million cells were injected into spleen of TK-NOG mice (CIEA) (n=10) or kidney subcapsular of NSG mice (Toronto) (n=10). All mice were housed under 12 light/ 12 dark cycle, temperatures of 22±2 Celusius with 50±10% humidity.

Wild animals

This study did not involve wild animals.

Field-collected samples

This study did not involve field-collected samples.

Ethics oversight

All animal experiments were carried out under procedural guidelines, severity protocols and with ethical approval from the Central Institute for Experimental Animals (CIEA) in Japan, and University Health Network Animal Care Committee (Toronto).

Note that full information on the approval of the study protocol must also be provided in the manuscript.

Flow Cytometry

Plots

Confirm that:

- [x] The axis labels state the marker and fluorochrome used (e.g. CD4-FITC).
- [x] The axis scales are clearly visible. Include numbers along axes only for bottom left plot of group (a 'group' is an analysis of identical markers).
- [x] All plots are contour plots with outliers or pseudocolor plots.
- [x] A numerical value for number of cells or percentage (with statistics) is provided.

Methodology

Sample preparation	Differentiated cells were dissociated into single-cell suspensions. Dead cells were excluded during flow cytometry analyses or DAPI staining. For cell surface marker analyses, staining was carried out in PBS with 10% FCS. For detection of intracellular proteins, staining was performed on cells fixed with 4% paraformaldehyde (PFA: Electron Microscopy Science, Hatfield, PA, USA) in PBS. Cells were permeabilized with 90% ice-cold methanol for 20 min for ALB, AFP, and CK7. Cells were subsequently incubated with secondary antibodies for 30 min at room temperature.
Instrument	Data collection was performed with BD LSRFortessa (BD Biosciences). Cell sorting experiments were performed with BD FACS Aria or Aria Fusion.
Software	FlowJo v10 was used for data analysis.
Cell population abundance	The purity of sorted population was checked by LSRFortessa from small amount of collected samples and ensured to be more than 95%.
Gating strategy	Cells were first gated based on light scatter properties based on cell size (FSC-A) and granularity (SSC-A), and width parameter<br>on foward scatter was used to gate out doublets. For marker expression, negative cells were gated based on isotype control (ALB, AFP, CK7), secondary only (DHIC5-4D9, HDE1) staining control. Cells stained with CXCR4, cKIT, EPCAM were classified based on unstained negative cells.

- [x] Tick this box to confirm that a figure exemplifying the gating strategy is provided in the Supplementary Information.
