# Supplementary material for: Generation of functional ciliated cholangiocytes from human pluripotent stem cells
Source: Nat Commun. 2021 Nov 11;12:6504. doi: 10.1038/s41467-021-26764-0 (PMC8586142; doi:10.1038/s41467-021-26764-0)
Supplement: Supplementary file 4 — Description of Additional Supplementary Files [file 41467_2021_26764_MOESM4_ESM.pdf]

Description of Additional Supplementary Files

File Name: **Supplementary Movie 1**
Primary cilia expression in hESC(H9)-derived cholangiocytes in monolayer culture.

File Name: **Supplementary Movie 2**
FSK induced swelling of hESC(H9)-derived cholangiocyte cysts.

File Name: **Supplementary Movie 3**
Secretin induced swelling of hESC(H9)-derived cholangiocyte cysts.

File Name: **Supplementary Movie 4**
ATP induced calcium signaling in GCaMP-derived 3D cholangiocyte cysts.

File Name: **Supplementary Movie 5**
TUDCA induced calcium signaling in GCaMP -derived 3D cholangiocyte cysts.

File Name: **Supplementary Movie 6**
ATP induced calcium signaling in GCaMP -derived plated down cholangiocyte cysts.

File Name: **Supplementary Movie 7**
Flow induced calcium signaling in GCaMP-derived cholangiocytes.

File Name: **Supplementary Movie 8**
Flow does not induced calcium signaling in non-ciliated GCaMP-derived cholangiocytes.

File Name: **Supplementary Data 1**
Differentially expressed genes in all hPSC-derived cholangiocyte clusters.

File Name: **Supplementary Data 2**
Differentially expressed genes in 2D dominant, 3D dominant and combine clusters.
